# Fibre Optic Sensors for Structural Health Monitoring of Aircraft Composite Structures: Recent Advances and Applications

**DOI:** 10.3390/s150818666

**Published:** 2015-07-30

**Authors:** Raffaella Di Sante

**Affiliations:** Department of Industrial Engineering—DIN, University of Bologna, Forlì 47121, Italy; E-Mail: raffaella.disante@unibo.it; Tel.: +39-54-337-4458

**Keywords:** fibre optic sensors, fibre Bragg gratings, Brillouin scattering, Rayleigh scattering, lamb waves, structural health monitoring, composite materials, smart structures, aerospace, aircraft

## Abstract

In-service structural health monitoring of composite aircraft structures plays a key role in the assessment of their performance and integrity. In recent years, Fibre Optic Sensors (FOS) have proved to be a potentially excellent technique for real-time *in-situ* monitoring of these structures due to their numerous advantages, such as immunity to electromagnetic interference, small size, light weight, durability, and high bandwidth, which allows a great number of sensors to operate in the same system, and the possibility to be integrated within the material. However, more effort is still needed to bring the technology to a fully mature readiness level. In this paper, recent research and applications in structural health monitoring of composite aircraft structures using FOS have been critically reviewed, considering both the multi-point and distributed sensing techniques.

## 1. Introduction

**Figure 1:**
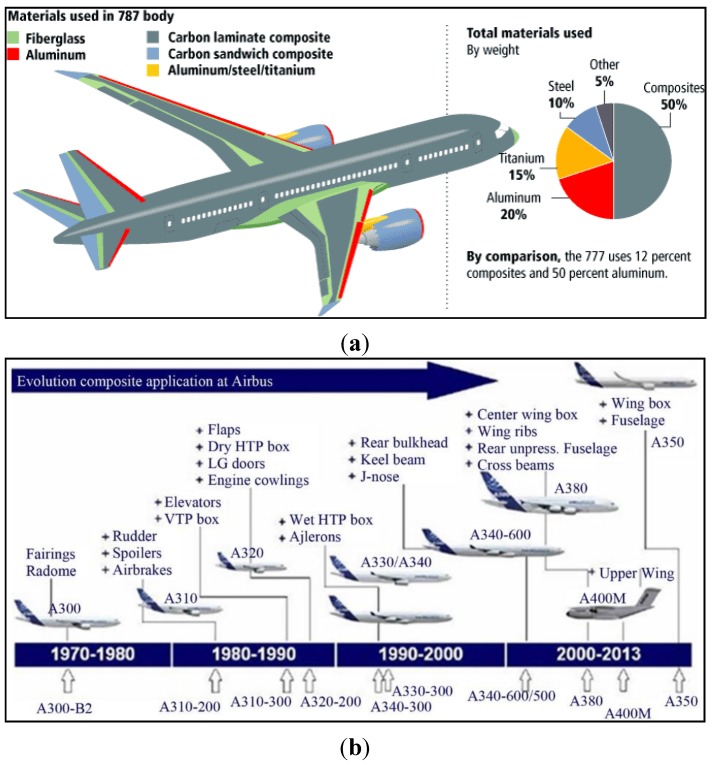
(**a**) Use of composite materials in the Boeing 787 and (**b**) evolution of the use of composites in Airbus aircraft

Today, high percentages of advanced composite materials are integrated into the primary flight structures of aircraft. For example, over 50% of the structural components of the Boeing 787 and Airbus 350 XWB are made of composite materials rather than conventional aluminium alloys. Figure 1 shows where composites are currently deployed in the Boeing 787 and how they have increasingly been replacing other materials in the development of new Airbus aircraft models.

Military airplanes such as the U.S. F-22, F-35 and F-117A fighters also make extensive use of composite materials. The increased use of fibre-reinforced materials is due to their light weight and superior strength properties with respect to conventional metallic materials, which make their deployment fundamental for the reduction of weight and hence the operational costs of aircraft [1]. Composite materials technology has become key to improving fuel efficiency, reducing emissions and lowering the manufacturing/operating/certification costs in current and future aircrafts.

Over the last two decades, the growth of air traffic has been impressive and will strongly increase in the forthcoming years, especially in the Far East. Due to the strategic importance of aviation in meeting societal and economic needs, Europe’s key stakeholders have developed visions for 2020 (Vision 2020) and towards 2050 (FlightPath 2050) [2]. Already by 2020 [3] it is expected that aircraft will be significantly more affordable, safer, cleaner and quieter than at the turn of the century. Specific targets have been identified; for example, CO_2_ emissions are to be reduced by 50%, NO_x_ emissions by 80%, accident rates by 80%, and the time to market halved. In this context, the use of composite materials is essential for the design of high-strength, lightweight aircraft structures, which may contribute significantly to the reduction of fuel consumption and pollutants without compromising flightworthiness. However, since composite materials are more complex than their metallic counterparts, durability and safety issues are more relevant. Their anisotropy and the fact that they comprise different materials (matrix and fibres) result in relatively unpredictable behaviour as concerns the initiation and propagation of damage and the estimation of the remaining strength and residual life of the structure. A significant amount of research has been conducted in order to investigate the influence of defects, especially those caused by impact, on the strength and life of composite structures, and to determine the critical size of damage. In parallel, much work has been done to develop non-destructive inspection/evaluation/testing techniques [4] useful for the structural health monitoring (SHM) of composite structures [5]. Particularly attractive in this area are intelligent monitoring systems that would enable continuous in-service load monitoring and damage detection of aircraft structures. Potentially such systems would enable the frequency and costs of maintenance on-ground inspections to be reduced, thus improving safety and reliability, and extending the operational life cycle of aircraft.

Several types of sensors have been investigated for SHM applications, including strain gauges, accelerometers, ultrasonic sensors, passive acoustic sensors, *etc.* Some of these sensors are expensive, difficult to deploy over large structures or unable to withstand harsh environments and to continue to operate for long periods of time. Among possible solutions for SHM, fibre optical sensors (FOS) [6], particularly those based on fibre Bragg gratings (FBGs) [7], have been emerging as an increasingly interesting technology due to their distinctive advantages which include higher sensitivity, immunity to electro-magnetic interference and durability. Furthermore, their multiplexing capability offers the possibility to reduce dramatically the cumbersome wiring required by electrical strain gauges and accelerometers, traditionally employed for load monitoring. Besides FBG-based measurement systems, fibre optic sensing techniques based on Rayleigh or Brillouin scattering of the light in standard telecom optical fibres have recently paved the way for truly distributed SHM systems, where the whole fibre acts as a sensor that is sensitive to strain changes with resolution in the centimetre or lower-than-cm range [8]. As specifically concerns damage detection, ultrasonic or acoustic piezoelectric sensors, considered as excellent candidates, are often likely to be in the development stage, to have a low multiplexing capability, lower durability and inferior strain-to-failure resistance.

For all these reasons, currently the scientific, industrial and end-user communities generally view fibre optic sensors to be the technology with the highest potential for continuous real-time monitoring of aircraft structures. The additional potential for integrating optic fibre sensors into composite materials during the layup process would also enable the monitoring of composite structures during their whole life cycle, improving their safety, reliability, cost efficiency and hence extending their operational life.

FBG-based sensors and measurement systems have already found a range of interesting practical applications in load and damage monitoring of aircraft composite structures, mainly in ground tests and design. However, some aircraft are already in operation with integrated networks of fibre optic sensors taking measurements during flight [9]. Airbus has recently reported that the long-term vision is that all new aircraft will fly with distributed FBG optical sensors [10]. Indeed, some of the developed solutions may already be considered relatively mature, *i.e.*, at a Technology Readiness Level (TRL) of 5–6. For example, FBG-based local damage detection has already proved viable and effective when applied to composite parts of aircraft such as bonded repairs [11]. Nevertheless the wider acceptance of the use of these sensing systems is still hindered by issues regarding sensor performance, especially when embedded, detection capability, maintainability, size and weight of the available interrogation equipment, and the lack of a standardisation and certification framework.

From the perspective of the ability to detect damage, one of the main challenges for both multi-point (FBG-based) and distributed sensing techniques remains the development of reliable methods to monitor key structural parameters related to damage inception and growth over large structures, with suitable physical and spatial resolution, even when the damage position is not known with sufficient precision a-priori. Over recent years, however, significant advances in the field of fibre optical sensors systems have produced innovative and powerful solutions, such as hybrid methods based on the simultaneous use of FBG and piezoelectric sensors [12] or hierarchical methods where the sensing architecture resembles the human nervous system [13]. These innovations may actually extend the potential of FOS-based SHM systems to the most challenging situations, such as damage detection in large composite structures.

This paper will firstly give an overview of the operating principles and available technologies for the interrogation of fibre optic sensors. Specifically, FBG-based and distributed fibre optic techniques which have already proved suitable for the structural health monitoring of aircraft structures, will be considered. Then, some of the most relevant issues regarding the deployment and use of fibre optic sensors in composites, such as mechanical coupling, fibre protection and the spectral response of embedded sensors, will be reviewed. Finally, the paper will present an extensive overview of the applications and related FOS-based SHM techniques that have been developed over the last decade for composite aircraft structures monitoring.

## 2. Operating Principles and Technologies

In the last two decades, the use of optical fibres and related optical components has dramatically increased, particularly in the telecommunications sector, thus enabling performance to be improved and costs to be reduced. This progress has facilitated the development of a large variety of fibre optic sensors, increasing the possibility of realising sensing technologies which are highly competitive with respect to traditional, less expensive electrical sensors. In particular, the cost of the sensing elements, such as fibre Bragg gratings, has decreased significantly over the last decade and, in the case of distributed sensing systems, even a standard, inexpensive telecom fibre can be used. However, the interrogation systems used to acquire and process the signals and retrieve the desired measurement information are still demanding in terms of complexity and cost.

The high interest and potential for practical application regarding fibre optic sensing technologies, confirmed by several recent market forecasts, is stimulating research directed at identifying more compact solutions which offer increased performance at a lower cost. At the same time, even if the technology is still relatively expensive, the benefit-to-cost ratio appears to be advantageous in cases in which potentially high volume applications are concerned, the structures to be monitored are complex and very expensive, and/or security and safety are a major issue. This is often the case in the aerospace industry, for example.

**Figure 2:**
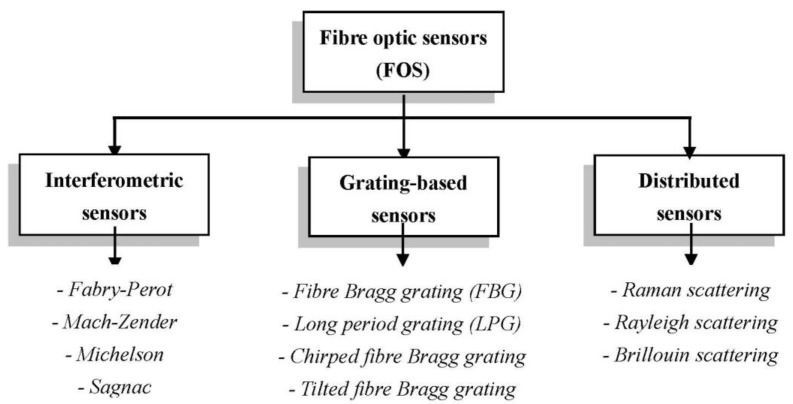
Overview of basic principles and types of fibre optic sensors.

Based on their operating principle, fibre optic sensors developed to date of interest for structural health monitoring can be categorised into three main types: interferometric, grating-based and distributed. Apart from being based on diverse measuring principles, these three types of sensors provide different spatially-resolved measurement capabilities. In general, interferometric sensors are suitable for single-point detection, while grating-based and distributed sensors can be used for quasi-distributed and distributed measurements respectively. Each category includes a variety of concepts that have been employed for different measurands and applications. Figure 2 gives a schematic overview of the major sensors types available.

The operation of interferometric sensors [14] is based on the change of the optical phase difference between two light waves with the same frequency, caused by the variation of a physical quantity. The major advantage related to the use of the interferometric principle is the high resolution that can be achieved which, for example, can exceed 1 µε for strain measurement. Sensors based on the Mach-Zender, and Michelson interferometers, also using long period gratings (LPGs) and photonic crystal fibres (PCFs) [14,15], have been used preferably for refractive index [16], temperature [17] or velocity measurement [18], while the Sagnac interferometer has been applied mainly to rotation measurements [19]. However, some of the first applications of fibre optic sensors for structural health monitoring, conducted by McDonnell Douglas in the 1980’s, used the Sagnac interferometer as a strain sensor [20]. Fabry-Perot and low coherent (SOFO) interferometric sensors are the most successful interferometric sensors that have been applied as yet to strain and strain-related monitoring applications. Fabry-Perot sensors [21,22] can attain a resolution as high as 0.15 µε and a measurement range that can extend to ±5000 µε. They are small with a length ranging from 1 to 20 mm and hence can be integrated into a structure without affecting its mechanical properties significantly; moreover, they can withstand temperatures of up to 250 °C. The major disadvantage related to the use of the mentioned interferometric sensors for structural health monitoring purposes is their low multiplexing capability, which limits their application to the measurement of a relatively low number of points. SOFO sensors [23,24] using low-coherence interferometry have been applied with success to a wide range of SHM applications, from bridges to oil pipes. Unlike Fabry-Perot sensors, SOFO are long-gauge sensors with lengths that are typically in the centimetre range while offering micrometre resolution. In addition, they are insensitive to temperature, highly precise and stable. However, their dynamic measurement range is quite low, only up to 1 Hz. This limitation makes them unsuitable for the detection of dynamic strains during operational loading, and for fatigue or the impact damage monitoring of aircraft structures.

Given their effective potential for exploitation in the aerospace, quasi-distributed and distributed techniques will be analysed in more detail in the following sections. In particular, fibre Bragg gratings and distributed techniques based on Rayleigh and Brillouin light scattering will be considered, due to the potential they offer for measuring both static and dynamic strains at a high number of locations. Besides outlining the operating principles, the technologies available for the interrogation of the fibre sensors will be briefly reviewed.

### 2.1. FBG Sensors

A fibre Bragg grating is a periodic modulation of the effective refractive index *n_eff_* that can be inscribed in the optical fibre core using mainly excimer lasers or UV sources and a suitable method for generating the spatial pattern, such as phase mask [25].

**Figure 3:**
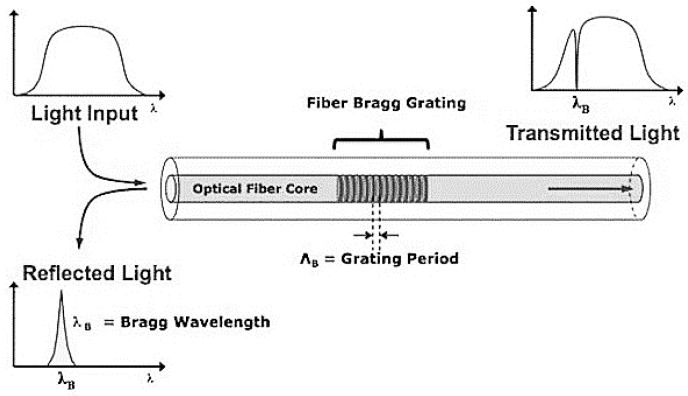
Fibre Bragg Grating’s principle of operation.

When a broadband light source is coupled to a single mode optical fibre with an inscribed Bragg grating, light travelling at the Bragg wavelength, λ*_B_*, a grating feature depending on the pitch, is reflected back by the grating itself and, as a result, is missing in the transmission spectrum. The principle of operation is illustrated in Figure 3. The reflected spectrum is centred on the Bragg wavelength and depends on the effective index of refraction and on the Bragg period (Λ_B_) of the grating, according to the following equation:

(1) $${\text{λ}}_{B}=2n_{eff}{\text{Λ}}_{B}\;$$

which is the Bragg equation.

When a local deformation is present, the grating’s period varies and the reflected wavelength changes accordingly, allowing detection of the local strain via the following equation:

(2) $$\frac{∆{\text{λ}}_{B}}{{\text{λ}}_{B}}=\left(1-{\text{ρ}}_{e}\right)\text{ε}$$

where *ε* is the longitudinal strain and *ρ_e_* the photo-elastic coefficient of the fibre core material. The term ρ*_e_* can be expressed as:

(3) $${\text{ρ}}_{e}=\frac{n_{eff}^{2}}{2}\left[p_{12}-\nu \left(p_{11}+p_{12}\right)\right]$$

where the *p_ij_* are the photo-elastic tensor components and υ the Poisson’s ratio.

For silica core fibres (ρ*_e_* = 0.22) and a grating with a central wavelength of 1550 nm, the typical strain sensitivity is approximately 1.2 pm/µm [26].

Strain and wavelength variations can be expressed via a one-to-one relationship only in the case of unidirectional deformation, provided that the effect of the temperature on the Bragg wavelength is suitably compensated. The wavelength shift related to temperature is obtained by differentiating Equation (1) with respect to temperature:

(4) $$∆{\text{λ}}_{B}=2\left(n_{eff}\frac{\partial {\text{Λ}}_{B}}{\partial T}+{\text{Λ}}_{B}\frac{\partial n_{eff}}{\partial T}\right)\text{Δ}T$$

It can be seen that a temperature increase causes a thermal elongation of the grating and therefore a change of the Bragg period and also of the refractive index. When the temperature change is small, Equation (2) can be re-written as [27]:

(5) $$∆\lambda_{B}=\lambda_{B}\left({\text{α}}_{f}+{\text{α}}_{n}\right)\text{Δ}T={\text{λ}}_{B}\beta \text{Δ}T$$

where $\alpha_{f}=\frac{1}{\text{Λ}}\frac{\partial {\text{Λ}}_{B}}{\partial T}$ is the thermal expansion coefficient of the optical fibre, which is approximately 0.55 × 10^−6^ K^−1^ for silica, and $\alpha_{n}=\frac{1}{n_{eff}}\frac{\partial {\text{n}}_{eff}}{\partial T}$ is the thermo-optic coefficient, which is dependent on the type and concentration of dopants.

Values between 3.0 × 10^−6^ and 8.6 × 10^−6^ K^−1^ for a germanium-doped, silica-core fibre have been reported in the literature [28]. The coefficients $\alpha_{f}$ and $\alpha_{n}$ can be combined in the temperature coefficient β. Linearity of Equation (5) holds only for small temperature variations, due to the fact that the thermo-optic coefficient is also temperature dependent. For higher values of the temperature, the equation becomes nonlinear and a polynomial interpolation is necessary.

Different solutions have been developed to compensate the sensor sensitivity with respect to temperature. The simplest method consists in measuring the temperature with an additional, strain free, FBG sensor [29], provided that both sensors are exposed to the same temperature. This method can be used also for embedded sensors, for example in composite materials [30]. When a strain free area is not available, one possibility is to encapsulate the reference FBG sensor in a suitable capillary [31]; in other methods, the compensating FBG sensor measures both the temperature and the strain, but the latter is different from that sensed by the measuring FBG sensor. The asymmetry may be due to an effectively different strain field [32,33], or to the use of another type of FBG sensor, e.g., with a distinct temperature-induced wavelength shift [34] or dopants [35]. However, these methods require a reliable measurement of the diverse strain field which is not always straightforward, especially for embedded FBG sensors. Methods also exist that allow intrinsic compensation of the temperature [36] or make use of temperature compensating elements attached to the fibre [37,38]. However not all of these are applicable in the case of embedded sensors.

**Figure 4:**
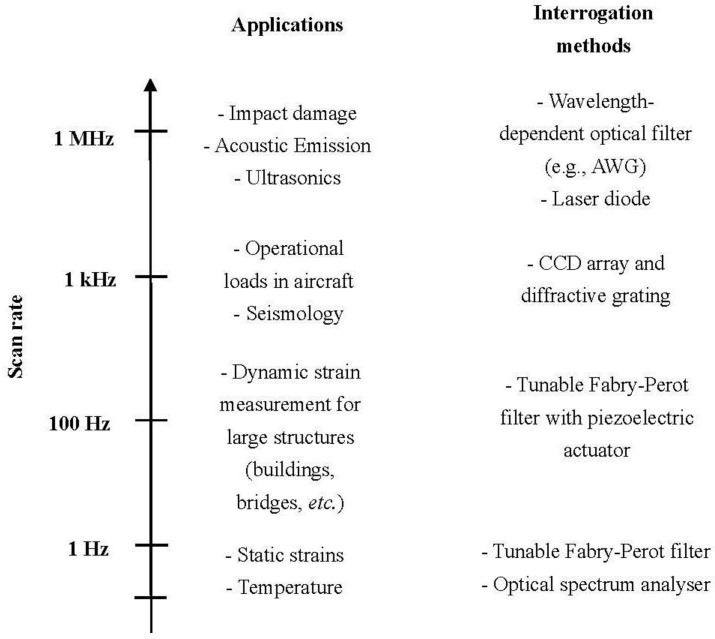
Schematic classification of FBG interrogation techniques based on scan rate and applications (adapted from [40]).

Over the past twenty years, a large variety of systems for the interrogation of FBG sensors have been developed. Interrogation aims at recovering the wavelength-encoded information from one or several multiplexed FBG sensors. Based on the scan rate as described in [39] and discussed in [40], the available interrogation techniques can be categorised as shown in Figure 4. In general, SHM of aircraft structures requires systems with a scan rate capability in the order of hundreds to thousands of hertz in order to capture phenomena ranging from operational deformations to impact damage [41]. For the measurement of strains under operating conditions, aimed at monitoring deviations occurring with respect to the predicted values in critical components and estimating fatigue life of the structural elements, required is a measurement range of thousands of µε and a relatively low scan rate. Instead, for the detection of damages due to impact or delamination, which can be very small and in a location not known a priori, in general it is necessary to use a high rate in order to exploit methods based on the detection of acoustic waves propagating in the material for example.

In order to be able to use a network of FBG sensors in a wide range of situations, the preferred choice would be a system with a scan rate variable in the required range. The scan rate should also be related to the high number of sensors that is usually required for high-spatially-resolved structural monitoring. Furthermore, in order to comply with usual requirements of aerospace applications, the interrogation system should be light, small, low-power consuming and immune to electromagnetic interference.

The interrogation systems that have been developed to date are usually suitable for different measurement frequency ranges, as shown schematically in Figure 4. The simplest available FBG interrogator is the conventional optical spectrum analyser (OSA), which is however cumbersome, costly and offers only low speed scanning. Below 1 kHz, it is possible to use tunable Fabry-Perot filters to realise wavelength sweeping by means of mechanical moving parts [42,43]. For measurements from 1 kHz to 20 kHz, a combination of a CCD array and a diffractive grating has proved effective [44]. Instead between 20 and 500 kHz, primarily three methods have been reported in the literature: the use of a laser diode with output wavelength centred in the middle of an FBG spectrum [45]; a Fabry-Perot filter with a broadened and fixed spectrum [46]; and an arrayed waveguide grating (AWG) with fixed spectrum [47]. Of these, the concepts of laser diode and arrayed waveguide grating are the most reliable [40]. If the laser diode is replaced by a tunable laser source [48], it becomes possible to operate the interrogator in the wavelength-sweeping mode for the measurement of strains due to operational loads. However, it is not feasible to operate the system simultaneously in the two modes. Instead, an AWG, the core device used for wavelength division multiplexing (WDM) in optical communication systems, can be used to overcome this problem; it has already been used successfully for the interrogation of FBG sensors [49,50]. Unlike systems based on a laser diode or a tunable laser source, the AWG-based system can achieve both high resolution and broad range, thus enabling both high speed and accurate monitoring of quasi-static strains. In addition, this type of system is lightweight and can be miniaturised.

Recently, Guo *et al.* [51] developed a wavelength interrogation system based on an Echelle diffractive grating (EDG), which deploys planar light wave circuits technology offering the same functionality as an AWG. The interrogation system based on EDG is capable of achieving a wavelength resolution of less than 1 pm with a measurement accuracy of ±10 pm and a scan rate of 300 kHz. Both low and high scan rate are provided, by using dedicated channels. In the “sweeping mode”, the transmission wavelength of the EDG channel is tuned by adjusting the EDG chip temperature allowing load monitoring, while in the “parked mode” the transmission wavelength of all EDG channels is fixed and can be used for damage detection.

Besides universities and research centers, a number of companies have also been developing novel interrogation systems, which in some cases are already available on the market. In particular, three novel solutions have been proposed recently by industry. In [52] Mendoza *et al.* describe a lightweight, high-speed and self-powered fibre optic sensor system based on the use of monolithic photonic integrated circuit (PIC) microchip technology in order to integrate and miniaturise the optical and optoelectronic components; reportedly the system is capable of measuring static and dynamic data over a range of ±4000 µε, at sampling frequencies of up to 500 kHz, therefore being suitable for the real time detection of operating load, fatigue and damages. Costa *et al.* [53] have developed a parallel processing interrogator that allows massive multiplexing of a high number of FBG sensors sampled at high rates. Groups of up to 16 sensors can be sampled simultaneously up to 1 MHz on a single fibre with the capability of switching between multiple fibres in the kHz range. Also in this case, the solution allows for smart composite sensing applications ranging from low-frequency load monitoring applications to ultra-high frequency acoustic emission (AE) monitoring and Lamb-wave based damage detection. In recent years, NASA and the company 4DSP LLC have collaborated on the design of a commercially available, quasi-distributed, fibre-optic-based system for gathering large amounts of data from massive multiplexing of FBG sensors at a high sampling rate with 1 µε resolution [54]; this instrument uses a tunable laser source and a Michelson interferometer to interrogate up to 64 separate sensors arrays, each comprising up to 2000 sensors located along a fibre cable with a length of up to approximately 24 m. The interrogation algorithm can support a 100 Hz sweep capability, making the system suitable for highly spatially resolved measurements of the aircraft structural shapes.

Finally, it is worth mentioning a recent strategy for FBG sensor interrogation that addresses the lack of robustness associated with multiplexing FBG sensors in one or more fibre arrays. Indeed, any damage to the fibre or optoelectronic components can hinder the full operation of the SHM system. In order to overcome this lack of redundancy, Wild *et al.* [55] developed a sensing architecture called Distributed Optical Fibre Smart Sensing (DOFSS) in which a large number of Smart Transducer Interface Modules (STIMs) [56] are positioned at the nodes of an all-fibre sensor network for the simultaneous distribution of sensing, power and communication.

### 2.2. Rayleigh and Brillouin Distributed Sensors

**Figure 5:**
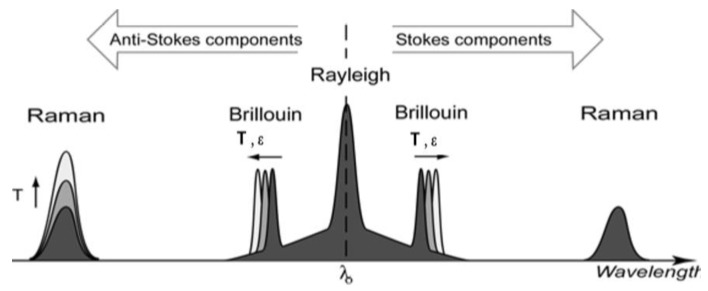
Typical spectrum of spontaneous light scattering in a fibre.

In distributed fibre optic sensing systems, the fibre itself becomes the sensor by detecting changes in the characteristics of the light scattered all along the fibre length caused by the local variation of physical quantities such as strain or temperature. Major mechanisms of elastic (*i.e.*, involving negligible energy transfer) and inelastic light scattering used in fibre optic sensing include Rayleigh, Brillouin and Raman scattering. Rayleigh scattering is an elastic physical phenomenon caused by non-propagating density fluctuations. The process is linear, meaning that the scattered light power is directly proportional to the input power. Since Rayleigh scattering is elastic, the related spectral component is centred at the frequency of the incident light in the typical spectrum of light scattered in an optical fibre (see Figure 5). Instead Brillouin and Raman scattering are inelastic physical phenomena and, as such, imply a degree of frequency shifting.

Brillouin scattering is due to the interaction of sound waves traveling in opposite direction. Two spectral components are associated with Brillouin scattering. The downshifted frequency peak is known as the Stokes component, while the up-shifted frequency peak is the Anti-Stokes component (see Figure 5).

Raman scattering is also an inelastic type of scattering and is due to interaction of the light wave with molecular vibrations in the transmission medium. The frequency of Raman peaks may oscillate due to fluctuations in the orientation of anisotropic molecules.

Each type of scattering mentioned is said to be spontaneous when there is no substantial modification of the optical properties in the propagation medium. On the contrary, when the light intensity is increased to a level which can cause modification of the medium properties, the scattering regime is said to be stimulated. A more detailed description of the various scattering mechanisms can be found in [8].

For strain measurements, Rayleigh and Brillouin scattering are of current interest, while Raman has been used mainly for temperature measurements [57]. Rayleigh and Brillouin scattering may be detected both in the time and frequency domains. The Optical Domain Time Reflectometry (OTDR) technique is based on time domain detection and can be used for both types of scattering. OTDR using Rayleigh scattering was initially developed for fault detection in the telecom field [58], based on the loss of light at the damage location. Different approaches to use this technique for sensing purposes have been reported in the literature, such as Polarisation OTDR [59], Coherent OTDR [60] and Phase OTDR [61]. However, these methods are limited with respect to practical SHM applications due mainly to uncertainty in measurement location and the need for a very large frequency bandwidth for the detecting electronic components in order to achieve millimetre resolution.

**Figure 6:**
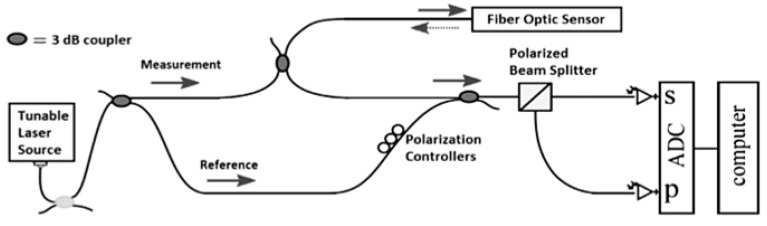
Basic OFDR network.

These limitations may be overcome by using Rayleigh scattering based Optical Frequency Domain Reflectometry (OFDR) [62]. Figure 6 shows a schematic diagram of a basic OFDR network. In a basic OFDR system, light generated by a tuneable laser source is split by a fibre optic coupler between a measurement and a reference path of an interferometer. The light reflected from the fibre optic sensor is recombined with the light in the reference path. The combined light passes through a polarization beam splitter and is collected by two detectors (marked with “S” and “P” in Figure 6). A Fourier Transform of the detected signals provides the amplitude and phase as a function of time delay along the fibre. Data obtained from the Fourier Transform is windowed around the specific sensor location. As a result, the length of this window becomes the actual spatial resolution achieved in the strain measurement. An inverse Fourier transform of the filtered data yields the spectrum of the specific fibre section. This spectrum is finally cross-correlated with the reference spectrum of the fibre section in the baseline state. The spectral shift obtained from this operation is converted to strain and/or temperature using the calibration factor. By repeating this process along the fibre length, it is possible to obtain a distributed measurement of strain [63,64].

The use of a tuneable laser source allows millimetre resolution over a fibre length less than 100 m. The coherence length of the light source limits mainly the sensing length in this type of system. Using a phase reconstruction scheme, the sensing length can achieve 1 km [8].

The first generation interrogators had scan and data transmission times of several seconds. The improvements in data processing, laser and laser driver performance have since enabled measurements at over twice the rate previously reported (approximately 250 Hz) [65]. However, the acquisition rate increase results in a larger spatial resolution and shorter sensing length.

The most performing systems [66] currently available commercially are based on the OFDR technique. They ensure a strain range of ±10,000 µε for temperatures ranging from −268 to +900 °C. The available combinations of sensing length, acquisition rate and gauge length range from 2 m, 250 Hz and 5.12 mm respectively, to 20 m, 50 Hz and 5.12 mm respectively. The accuracy is of ±5 µε in the first case and ±10 µε in the second. The lowest gauge length achievable is 1.28 mm with an accuracy of ±20 µε. In this case, the acquisition rate decreases to approximately 23 Hz and the sensing length is of 10 m. For quasi-static measurements (2.5 Hz), the highest sensing length is 50 m, with a spatial resolution of 1 mm and accuracy of ±2 µε.

Although the Brillouin scattering signal is up to 20 dB lower than the corresponding Rayleigh signal, its detection is based on the frequency shift of the Brillouin spectral components, rather than requiring a cross-correlation operation. This type of measurement is absolute if compared to the differential measurement performed by the OFDR-based system. Also, Optical Time Domain Reflectometry (OTDR) systems based on Brillouin scattering have a sensing length greater than 150 km. Such an advantage makes Brillouin distributed sensors potentially extremely attractive for monitoring large structures. This is the main reason why much research has focused on this type of system over the last two decades [67]. Distributed sensors based on Brillouin scattering were initially developed considering spontaneous scattering. Brillouin Optical Time Domain Reflectometry (BOTDR) [68] allows static strain measurements with accuracy of 60 µε and gauge length of 1 m, up to approximately 50 km. Instead Brillouin Optical Time Domain Analysis (BOTDA) is based on the use of stimulated Brillouin scattering and can achieve a spatial resolution of 1 m [69]. The first Brillouin scattering sensor system which had a gauge length of less than one meter (50 cm) was developed in 1998 [70] and was further improved to achieve a spatial resolution of 10 cm [71] by using a spectrum de-convolution technique. Later, innovative approaches such as the use of pre-pumping [72], differential Brillouin gain [73] and Brillouin echoes [74], have lowered the spatial resolution down to 2 cm over 2 km, with accuracy of 20 µε. Pre-pump pulse and Differential pulse width pair BOTDA (PPP-BOTDA and DPP-BOTDA, respectively) have therefore achieved a sensing length comparable to that reached by techniques based on the frequency domain detection (OFDR) of Rayleigh and Brillouin scattering. However, OFDR-base systems are limited to a sensor length of below 100 m. Brillouin scattering based OFDR techniques consist mainly in the Brillouin Optical Frequency Domain Analysis (BOFDA) and Brillouin Optical Correlation Domain Analysis (BOCDA) systems. BOFDA [75] features have been improved from an initial spatial resolution of 1.4 m over 11 km, to 3 cm over a sensing length of 9 m [76]. BOCDA [77] is able to provide a gauge length of 1 cm for sensing length in the tens of meters range [78] and 7 cm over a range of 1 km [79]. Unlike Rayleigh scattering sensors based on the frequency domain detection, which have recently proved capable of dynamic measurements up to 250 Hz, Brillouin scattering sensors are mostly used for static measurements.

Currently available commercial systems [80] are based on the PPP-BOTDA technique. They offer a spatial resolution of 10 cm with accuracy of 4 µε, over a sensing length of more than 1 km. A less accurate version of the system (15 µε accuracy) reaches 2 cm spatial resolution over 0.5 km. Hybrid Rayleigh/Brillouin systems are also available [80] which provide an accuracy of 0.5 µε for gauge length of 5 cm over 1 km, or gauge length of 2 cm over 0.5 km.

## 3. Sensor Deployment and Performance in Composite Structures

The installation of FBG sensors in composite structures for structural health monitoring requires measures and techniques to guarantee their reliability and durability. Usually the sensors are surface-mounted or integrated into the composite structure. Surface mounting is less challenging than embedding but also less suitable, due to the high fragility of the optical fibre. Much research has been reported in the literature regarding the strain transfer between surface-mounted FBG sensor and substrate. In this case, the adhesive layer thickness and mechanical properties play an important role on the transfer of strain to the fibre core [81]. In [82] it was found that the most important factors are the adhesive thickness and the length of bonding.

Due to their small size and flexibility, optical fibres can be embedded in composite structures during the manufacturing process. The advantages are the enhanced protection of the fragile fibre sensor and the possibility of strain monitoring and damage detection in different locations inside the material. However, sensor integration in composites presents a number of issues and shortcomings that need to be addressed properly. Some of the most relevant among them will be discussed briefly in the following sections.

### 3.1. Mechanical Coupling

**Figure 7:**
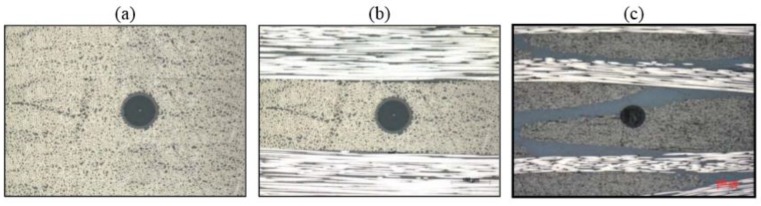
Optical fibre section in (**a**) unidirectional; (**b**) cross-ply and (**c**) woven fabric [83].

Despite being very small, a standard telecom fibre has a size (125 µm) which is much larger than commonly used composite fibres (e.g., 5–10 µm for carbon fibres). As a result, resin rich regions are created around the fibre, especially in the woven fabric. Figure 7 shows clearly the comparison between the sizes of the sensor and the host material for different types of fabrics [83]. Even with the small diameter (50 µm) fibres that have been developed for sensing purposes in aerospace structures [84], the difference is still of an order of magnitude. However, it has been demonstrated that small diameter fibres do not produce any significant modification of the mechanical properties in the host structure [85]. Standard telecom fibres shows a similar minimally intrusive behaviour when embedded parallel to the reinforcing fibres [86].

### 3.2. Fibre Protection at Ingress-Egress Points

**Figure 8:**
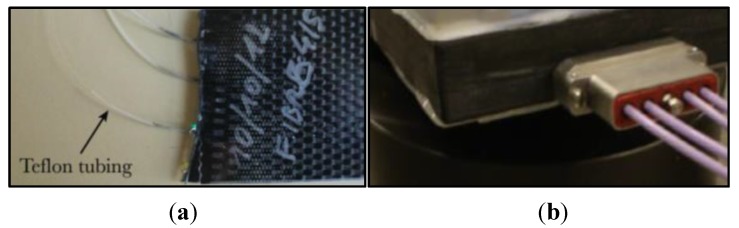
Teflon tubes (**a**) [94] and embedded connector (**b**).

Ingress and egress points of the fibre in the material are critical locations due to the sharp pressure gradient and therefore the severe bending experienced by the fibre. The concentrated stress acting during some of the manufacturing processes, such as the vacuum bag technique, and later under operating conditions, cause severe optical loss [87], and may lead to fibre breakage. Different approaches have been reported in the literature to mitigate this problem. The simplest method consists in the use of small plastic tubes [88,89] protecting the fibre along short sections inside and outside the material (see Figure 8a). The tubes are usually made of polytetrafluoroethylene material (PTFE, also called Teflon) or polyvinylidene fluoride (PVDF), and may be reinforced by using a metal coil [90]. Moreover, their openings must be sealed to avoid the ingress of the resin. In order to allow cutting and polishing of the surface after manufacturing, the fibre ingress and egress points should be located before the end section of the composite structure [90]. In this case, care must be taken to avoid sharp curvature of the fibre and thus prevent high optical loss. However, when the fibre is embedded below several plies this method is not applicable.

Partially embedded (also called “surface-mounted”) or embedded connectors offer another possible solution. In the first case, the connector can be positioned on one of the surfaces of the composite structure [91]. A miniature connector of this type is presented in [92]. When the fibre is embedded between internal plies, this solution is also difficult to implement. Embedded connectors [93,94,95] are specially designed devices to overcome this problem; only the female part of the connector is embedded so that proper machining of the composite element surface is still possible (see Figure 8b). These connectors are often miniaturised [94] to be less invasive. However one potential source of decreased reliability is the deformation that the embedded connector might experience during the manufacturing process which may affect the alignment [95]. In addition, the use of embedded or partially embedded connectors requires a careful assessment of the potential reduction in the strength of the composite.

Most of the mentioned problems can be avoided by using the so-called free-space coupling [94], which enables coupling of free-space light into and out of an embedded FBG sensor without the use of a physical connector. This result is obtained by splicing a multimode fibre to a single mode fibre Bragg grating, and using hand polishing to integrate 45° mirrors into the ingress and egress point of the fibre and the grating respectively. Although the estimated total loss was quite high (23 dB), by using this technique it was possible to measure accurate strain values up to 2000 µm and increase system robustness and simplicity.

Finally, during composite part manufacturing it is important to protect the outer section of the fibre from excess resin that may escape from the layers pile.

### 3.3. Spectral Response in Embedding Process

The spectral sensitivity of an embedded fibre optic sensor can deviate from that of the non-embedded sensor for a number of reasons. During the heating phase of the manufacturing process, the fibre is subjected to the stress created at the boundary between the coating and the composite ply; however, during the cooling phase, this stress is only partially released. This results in residual strains acting inside the material and on the fibre. Consequently, the sensor spectral response is affected by the existence of transverse, and often non-uniform, strain fields.

In the literature, various works have addressed this issue. In [96] it was observed that the spectrum of two embedded FBG sensors was severely distorted after the manufacturing process. The Bragg wavelength peak split into two spectral components, due to the existence of transverse strain. This effect is attributed to birefringence of the optical fibre and the difference between the peak wavelengths may be used as a measure of the transverse strain. However, such a distortion of the FBG spectrum prevents measurement of the longitudinal strain, based for example on the commonly available algorithms for Bragg wavelength detection, such as the Full Width Half Maximum (FWHM) method. Even if the algorithm were used to track both peaks and FBG sensors were added for multi-axial measurements [97,98], it would be necessary to distinguish the two spectral components of regular shape. That is not always the case, as transverse strain or local strain gradients can cause severe distortion of the spectrum [99,100]. The fibre coating plays, however, an important role in minimising the effect of transverse strain due to the manufacturing process. When an acrylate or polyimide coating are used, the Bragg wavelength is simply shifted due to longitudinal transverse strain, but the spectrum is not distorted [90,101]. Even in this case though, the accurate measurement of pure longitudinal strain with a uniaxial FBG sensor may be undermined depending on the load amount, type and point of application [90,100,102]. For all these reasons, and considering also that the strain is transferred across the coating to the fibre core, accurate calibration of the embedded sensors after the manufacturing process and critical evaluation of the actual operating conditions are mandatory to ensure the reliability of the measured data.

When it comes to damage detection rather than simple strain monitoring, the information given by the distorted spectrum of bare embedded FBG can be quite useful [103,104]. By relating the distortion to the specific defect and damaging mechanism, it is possible to detect cracks, delamination or debonding in various types of composite laminates [104]. A similar method to detect impact damage can be used with distributed sensors [105]. In those cases, the use of optoelectronic demodulators with full spectrum detection capability is needed. In addition, the fibre optic sensor must be in close proximity to the damage location, requiring an a-priori estimation or knowledge of the defect position.

## 4. Applications of FBG Sensors

### 4.1. Strain-Based Deformation Shape Reconstruction

Deformation shape prediction plays an important role in structural health monitoring and control of aerospace structures. Estimation of the deflection shape of a wing during flight enables in-flight monitoring of various lightweight aeroelastic systems, such as flexible flying wings or unmanned air vehicles (UAV), and commercial aircrafts. Fiber Bragg gratings can be applied beneficially to these structures instead of electrical strain gauges in order to obtain strain measurements useful for shape-detection algorithms.

Various studies have been reported in the literature regarding the estimation of displacement starting from strain measurement. One possible approach consists in the use of an inverse finite element method that relies on a least squares variational principle to predict the deformation shape of 3D plate and shell elements from strain measurements [106]. The application of this approach to SHM of 3D structures is reported in [107]. A frequently used approach is a modal-based algorithm that uses the mode shapes of the structure to transform the measured strains into displacements [108]. Several authors have used this modal-based algorithm and strain measurements obtained by means of FBG sensors for shape reconstruction. Kang *et al.* [109] and Rapp *et al.* [110] used measured strain data and strain-to-displacement transformations to estimate dynamic structural displacements, Rapp *et al.* [111] estimated the displacements of a 2D structure using its mode shapes, and Kim *et al.* investigated shape reconstruction for rotating structures [112].

**Figure 9:**
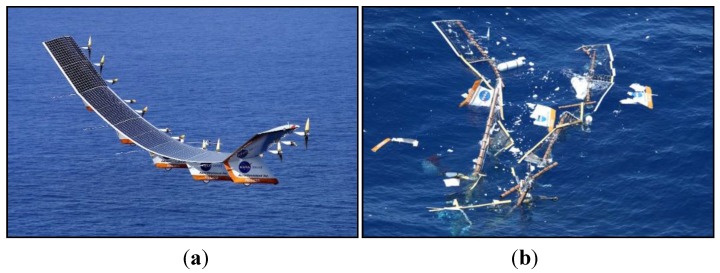
NASA’s Helios wing (**a**) and its wreckage in the Pacific (**b**) (courtesy of NASA).

Following the in-flight break-up of NASA’s Helios wing in 2003 (see Figure 9) and the awareness that part of the failure was most probably due to excessive wing deformations, the NASA Dryden Flight Research Center (DFRC) felt the urgency to develop a robust shape-detection methodology. The Helios Prototype, a solar-powered “atmospheric satellite” with a wingspan of 75.29 m (larger than that of the Boeing 747), was made mostly of composite material. The main tubular wing spar and the wing ribs were made of epoxy and carbon fiber. As a result, at DFRC a new monitoring technique was proposed based on the use of FBG sensors to acquire strain data in real time and a computationally efficient algorithm able to estimate accurately the deformation field from the measured data [113,114,115].

**Figure 10:**
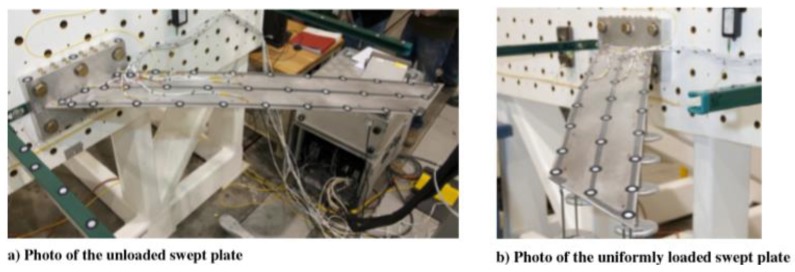
Photos of the wing-like swept plate experiments with three lines of FBG sensors (property of the NASA Dryden Flight Research Center) [116].

In [116] Derkevorkian *et al.* analyzed the method, compared it to the modal-based approach and applied it to a cantilever swept wing-like plate, which is shown in Figure 10. Three fibers with 100 FBG sensors each (spaced by 12.7 mm), were placed at the top and bottom surfaces of the plate, and a series of white markers was used to acquire photogrammetry images.

The rectangular plate was mounted at a 45 degrees angle from the test plate and subjected to different loading conditions in order to simulate a wing representing a variety aerospace structures. In particular, three loading cases were considered by applying point loads: (1) only at the trailing edge; (2) uniformly (see Figure 10b) and (3) only on one point at the plate tip.

A finite element (FE) model was created to represent the plate and the different loading conditions. In order to estimate the deflection shape, the FOSS (fiber optic strain sensor) algorithm was developed so as to use the strain data measured directly by FBG sensors. The algorithm relies on the classical beam theory to derive the displacement transfer functions in segments of the structure surface; details can be found in [113,114,115]. The approach is therefore different from the modal-based approach in that the latter derives the displacement-strain-transformation matrix from the displacement and strain mode shape matrices. In this case, the number of measured data can be less than the estimated displacements, which corresponds to the numerical mode shapes. This means that with a relatively few strain measurements, in principle a large displacement field can be obtained.

The estimated error was calculated by comparing this set of data with the displacements from the FE model. In the FOSS algorithm, the deflection shape was compared instead with the results from photogrammetry. The rms error value between the FOSS estimate and photogrammetry results was below 2%, while the average normalized error with the FE analysis displacements was between 2 and 3.7%. This difference can probably be attributed to modeling errors, which can be more serious in the case of complicated structures such as a real composite wing. The error can also be reduced by increasing the number of modes, but this would require a greater computational effort. Furthermore, the advantage related to the need for a few sensor locations to obtain the displacement information over a large area has become less important following the advent of optical strain sensors, which can be used in large numbers on lightweight structures. This opportunity also decreases the possible adverse effect of a damaged or inaccurate sensor.

Overall, an algorithm based on a large number of measured strain information obtained from FBG sensors has the advantage of being relatively independent from modeling errors and requires less computational effort in the case of real-time monitoring of high-aspect-ratio aircraft wings, to which the classical one-dimensional beam theory applies.

### 4.2. Strain Monitoring in Wing Structures

**Figure 11:**
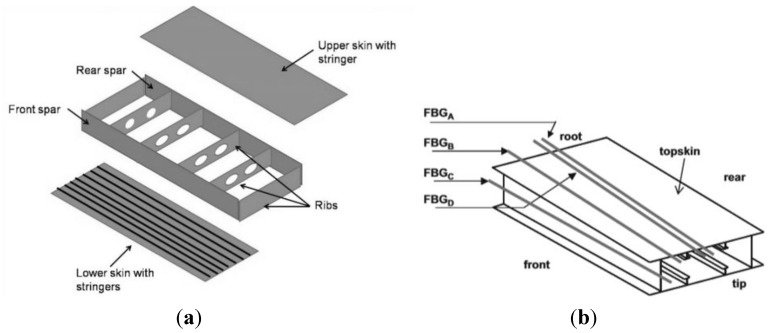
Typical scheme of wing box (**a**); deployment of the optical fibres in the structure (**b**).

Currently composites are used mainly for load-bearing primary structures such as the wing box, which is a structural component with “skins” comprising the top and bottom, and the front and back formed by I-beams (“spars”), interior stiffeners (“ribs”), and in-out stiffeners (“stringers”) (see Figure 11a).

Recently structural health monitoring systems based on fibre optical sensors have been developed to monitor dynamic strains in a wing spar under wind tunnel conditions [117] or in the wing leading edge during flight [118]. In 2008, Ryu *et al.* [119] instrumented a wing box using 24 FBG sensors and an interrogator based on a wavelength-swept fibre laser (WSFL) [120,121], demonstrating that this type of interrogation system, together with fast signal processing techniques based on signal digitalization, is able to monitor in real-time the buckling behaviour of a wing box. Three sensor arrays, each consisting of 6 gratings, were embedded in the top skin, and one array with 6 gratings was embedded in the front spar of the wing box, as shown in Figure 11b. In order to better monitor the overall behaviour of the wing, three out of the 6 gratings in an array were embedded below the outer external layer and the remaining three below the outer inner layer of the composite panel. Each sensor array was included in one optical fibre; hence, the system consisted of four optical fibres in total. Optimal location of the optical fibres and gratings was determined based upon a previous finite element analysis aimed at highlighting the areas which are most sensitive to buckling, *i.e.*, where the strain change was most pronounced. The instrumented wing box was then subjected to an up-bending test in order to simulate the lift-induced bending of a wing during flight. When buckling actually started in the front spar and afterwards, during the test, in the top skin near the clamped edge of the wing box, the corresponding FBG sensors were able to detect it in real-time in good agreement with the electrical strain gauges used as reference at the same locations.

Due to the capability of simultaneously gathering data from a high number of sensors through multiplexing technologies and fast processing techniques, in general a system of this type would be suitable for full-scale detection of relatively slow dynamic processes such as buckling induced by operating loads in large composite structures.

Instead a previous study focused on the durability and damage tolerance of a composite wing structure [122]. In particular, the authors used a small specimen simulating a portion of the upper wing skin of a small-size jet which was instrumented with FBG and acoustic emission (AE) sensors. To verify durability, the authors introduced impact damages and planned subsequent fatigue tests. Finally a static load was applied up to the design limit load in order to estimate the residual strength. The data from the FBG sensors was measured by a commercial fibre Bragg grating interrogator based on a wavelength-swept laser, capable of monitoring up to 256 gratings at a frequency greater than 100 Hz. After the impact which caused a barely visible impact damage (BVID), a variation was observed in the spectrum shape of the FBG sensor placed exactly below the impact point but on the opposite face of the specimen, which became broader and distorted. This result, due to the propagation of cracks perpendicular to the fibre grating, had been already documented in the literature [123,124]. During the fatigue tests, the FBG sensors allowed monitoring of the strain values in the measurement locations close to the impact points which were found to be in good agreement with those measured using reference electrical strain gauges. Using AE sensors during the tests and comparing the results with the images obtained via Pulsed Thermography and Ultrasonic C-Scanning, it was discovered that the impact damages did not progress. Indeed FBG sensors, while demonstrating their capability for long-term structural health monitoring, did not indicate any significant change in strain.

**Figure 12:**
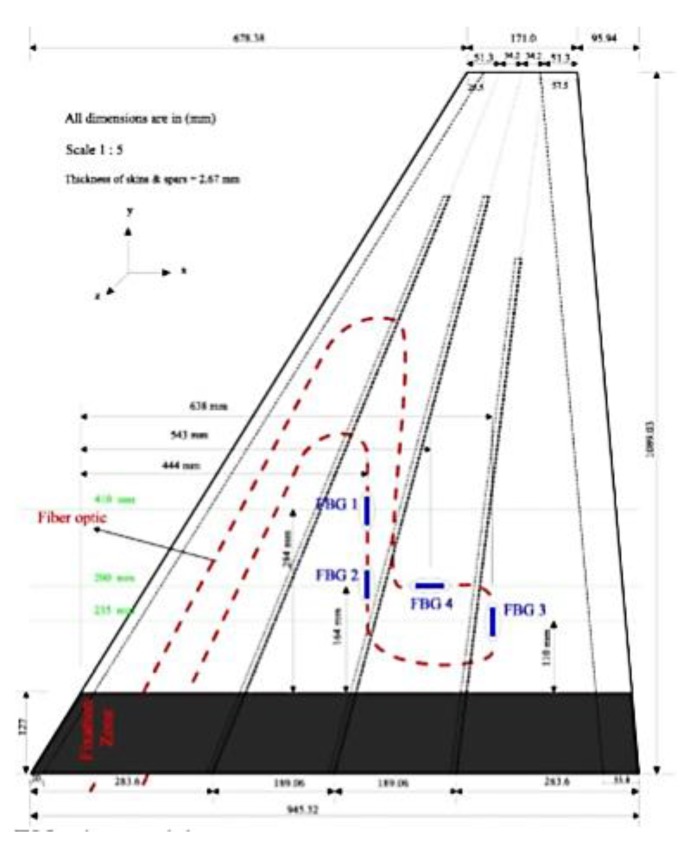
T38 wing model with the embedded FBG sensors [53].

More recently, as already mentioned in the section on FBG interrogation systems, Costa *et al.* [53] reported on a newly developed parallel processing interrogator that allows massive multiplexing of FBG sensors sampled at high rates, capturing loading and relatively low frequency vibration phenomena; the T38 wing quarter scale model shown in Figure 12 was sensorised and results obtained for sampling of four sensors at 6 kHz. The FBG sensors were embedded under the top layer of the composite model wing, in positions selected based on the results of an FE analysis. In particular, the FBG sensors were placed in zones of local high strain concentration but low spatial strain gradient. As explained in Section 3, the latter could in fact distort the Bragg spectrum. Both static and dynamic tests yielded precise results.

### 4.3. Life-Cycle Monitoring of L-Shaped Parts

L-shaped parts used in complex aircraft composite structures as wing spars, for example, are a critical element due to spring-in distortion introduced during manufacturing and through-thickness tensile stress: Spring-in distortion is due to the anisotropic nature of laminated composites and can cause problems in assembly when the part is forced to achieve the desired geometry, resulting in the accumulation of stresses; in addition, the sharp curvature of L-shaped components causes through-thickness radial stress to build up under operating conditions.

In [125,126] Minakuchi *et al.* developed a FBG-based life cycle monitoring system for L-shaped corner parts, embedding optical fibres in the corners of an L-shaped composite specimen in order to monitor the cross-sectional strain change during the whole structural lifecycle. Based on the berefringence effect of an embedded FBG sensor, it was possible to retrieve the cross-sectional strain history during cure, demolding and a four-point bend test which simulated operating conditions. The FBG sensors were able to capture internal deformation stresses during cure and demolding, offering an indirect measure of the corner angle and hence also a valuable tool for quality inspection of L-shaped composite parts. During the four-point bending test to simulate operation of the part, the embedded sensors could continuously monitor the non-axisymmetric strain change until failure due to crack propagation in the corner part. In this test, the through thickness tensile strain arising at the specimen sharp curvature was clearly predominant. When longitudinal stress is not negligible, the method should be improved either by adding another FBG sensitive only to strain in the longitudinal direction or by modifying the microstructure of the FBG embedded in the corner in order to detect transverse strain only [127].

### 4.4. Detection of Debonding in Composite Patches and Lap Joints

**Figure 13:**
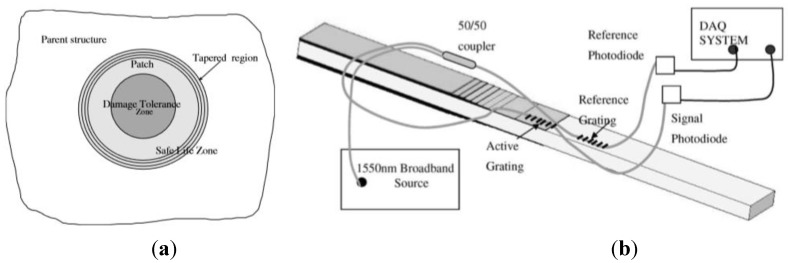
Safe life or damage tolerant areas of the composite patch (**a**) and SHM system for monitoring composite repair disbond with FBGs (**b**) (adapted from [11]).

Another area of interest for FBG-based intelligent systems is the repairing of cracked metallic airframe structures with adhesively bonded composite patches. Although efficient and cost-effective, the use of bonded composite patches is affected by the difficulty in predicting the long-term durability of adhesive bonds to metallic structures. In [11] the authors propose a simple strain-based SHM approach for monitoring the boron/epoxy patch repair of a critical fatigue crack in an F-111C wing, focusing on the area most exposed to environmental damage and fatigue, *i.e.*, the tapered edges of the patch undergoing the highest stress (see Figure 13a) for the purpose of an effective continuous SHM of the bonded repair.

The strain-based approach was developed during the realisation of a boron/epoxy doubler to reinforce the steel wing pivot fitting of Australian Defence Force (ADF) F-111C aircraft [128], where a crack was discovered during a routine visual inspection. Initially the SHM system was based on the use of surface-mounted electrical strain gauges. All sensors were located in positions where non-destructive inspection detected small disbonds, occurring after a service period and fatigue test. During a further fatigue test, results showed that strains in the patch edge decreased, revealing the growth of the disbonds in this region. However, some of the sensors showed anomalous behaviour (increasing rather than decreasing measured strain, probably due to the bonding of the sensor over an already disbonded region) with some failing close to the end of the test. In 2006 a similar strain-based approach had been used for a boron/epoxy repair on a F/A-18 inboard aileron hinge [129]. In this case, piezoelectric film sensors were used instead of electrical strain gauges due to their good fatigue durability and self-powering capability. Conventional and piezoelectric strain gauges may not be the optimal choice, however, considering their limited fatigue and environmental resistance and the need for multiple single gauges or films; thus fibre Bragg gratings were evaluated as possible alternative strain sensors, based in particular on advantages such as multiplexing capability, durability and greater strain-to-failure. The authors developed a modified sensing arrangement with two FBGs, a coupler and two photodiodes, as shown in Figure 13b. One of the two FBGs, referred to as the reference grating, is mounted in a region with no thermal residual strain, while the second FBG, namely the active grating, is bonded to the tapered region of the repair.

This arrangement allows compensation of temperature fluctuations and minimisation of possible strain gradients due to bending. The relative wavelength shift causes a change in the output optical power, which is measured using a high-speed, lightweight photodiode. When the repair begins to disbond, the active grating expands due to thermal residual strain release and transmits light as a result of the mismatch with the unchanged reference FBG wavelength. During a fatigue test of specimen, FBGs survived 700.000 cycles at strains of up to 3150 µε while the electrical strain gauges used as a reference failed. Overall, the strain ratio technique proved to be robust, low-cost and suited to the miniaturisation of the opto-electronic components and to embedding of the sensors directly into the tapered region of the repair.

**Figure 14:**
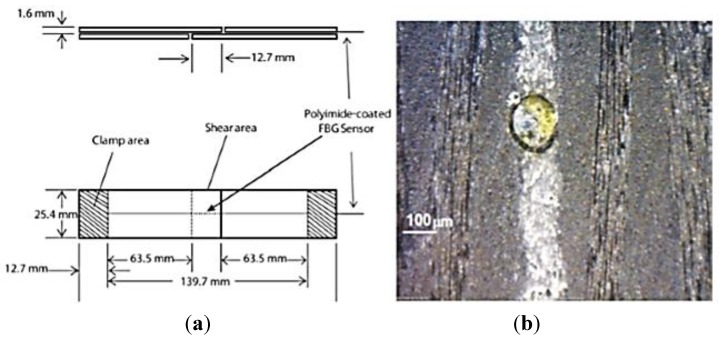
(**a**) Specimen with composite lap joint and embedded optical fiber; (**b**) micrograph of an embedded FBG sensor [132].

The maintenance and inspection of adhesively bonded composite lap joints are also challenging activities, since these joints cannot be disassembled like bolted metallic joints. In-flight or between-flight inspections monitoring of the joints could improve the description of their conditions and therefore enable the detection of premature failure. Direct measurements of the joint condition can be performed by embedding sensors into the adhesive layer, provided that these sensors do not affect the performance of the joint. Fiber Bragg gratings were applied for this purpose in [130,131], where cracking of the adhesive layer was observed through the distortion of the FBG spectrum, due to resulting local strain gradients. However, these works were performed under static conditions and therefore without taking into account the sensitivity of the joint structural dynamics to damage during in-flight conditions. In this case, distortion of the FBG dynamic response involves nonlinear phenomena and more complex strain fields arising in the composite joint and, above all, requires sufficiently high data acquisition rates to be captured. In [132] Webb *et al.* applied a recently developed dynamic full-spectrum FBG sensor interrogator [133] to investigate the spectral response of the FBG sensor embedded in a lap joint during dynamic loading. The specimens used for the experiments consisted of four adherends fabricated using 2 × 2 twill woven carbon fiber prepreg and joined using a structural aerospace paste adhesive. A polyimide-coated optical fiber with a 10 mm long grating was integrated into the adhesive layer during the joining process, as shown in Figure 14.

The composite lap joint specimens were subjected initially to cyclic loading in order to induce fatigue damage and then excited using a multicomponent harmonic excitation frequency range representing that of a typical in-flight load spectrum. During the fatigue test, the dynamic response of the joint became nonlinear and then potentially chaotic, as the fatigue-induced damage progressed.

**Figure 15:**
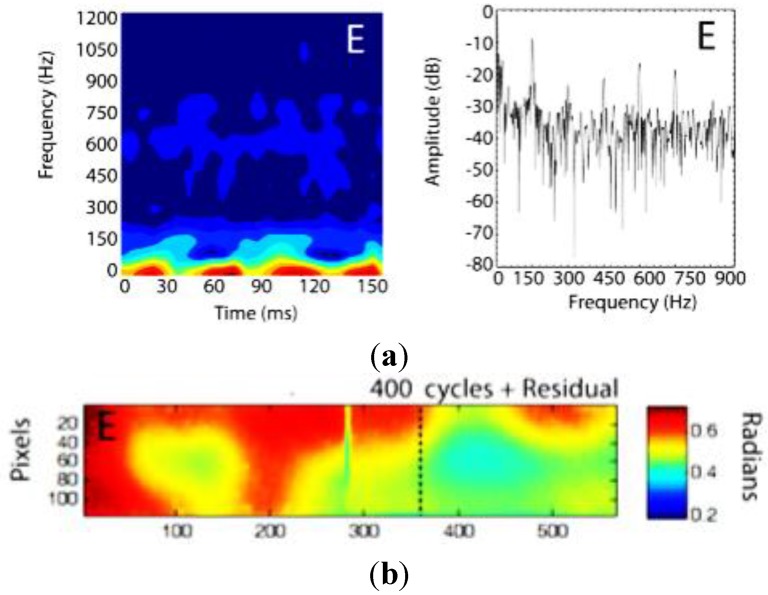
(**a**) STFT (left) and FFT (right) computed from the FBG peak wavelength estimation during fatigue tests; (**b**) corresponding pulsed-phase thermography phase angle image [132].

In parallel, the FBG dynamic response changed accordingly to damage growth, as could be seen using thermal imaging and a finite element model of the joint. Figure 15 shows the short-time Fourier transform (STFT) and the fast Fourier transform (FFT) of the time variation of the peak wavelength values extracted from the dynamic, full-spectral measurement. The FBG intensity spectra were collected at an acquisition rate of 100 kHz with a wavelength resolution of 84 pm. Figure 15 also depicts a pulsed-phase thermography phase angle image showing the fatigue-induced damage zone (indicating delamination or poor bonding) progressing to the FBG position.

### 4.5. Impact Damage Detection

The detection and localization of impact is of the highest importance in composite aircraft structures monitoring. This is a major concern due to the mechanical strength of the composite outer skins which can sustain a large deformation without developing cracks, even though the internal sub-structure is damaged. As a consequence, in some cases this type of damage can be undetectable during visual inspections.

**Figure 16:**
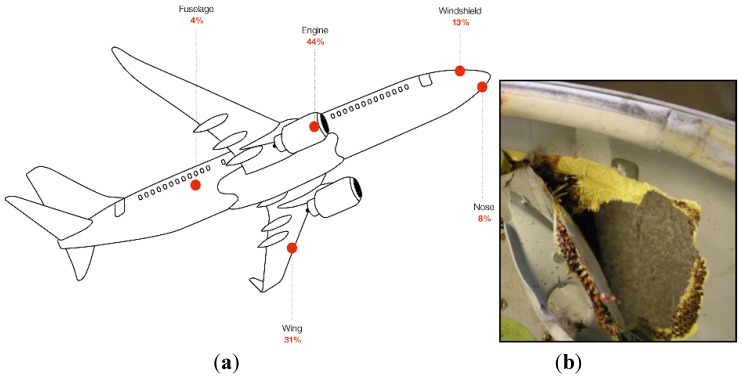
Bird strike impact probability (**a**); impact damage in a flap fairing of a cargo B767 caused by bird strike (**b**).

The most likely causes of aircraft impact damage during operating conditions include bird strike, hail, blade-off and lightning. Figure 16a shows that three quarters of bird strikes involve the wings and engines, but that potentially any part of an airplane may be concerned. Among the strategies to reduce the consequences of a bird strike, flight crews should be provided with useful information to detect bird strike while in flight in order to take appropriate action and maintain control of the airplane. Bird strikes can indeed cause severe damage, as shown in Figure 16b. Damage may also occur during ground service operations. Ground service equipment, such as ground vehicles or cargo loaders, account for a major percentage of damage occurring to commercial aircraft. Low-velocity impact caused by dropping tools generate barely visible impact damage (BVID) of a specific dent depth. Blunt impacts can result in severe damage to internal stiffeners, such as fracture or separation from the skin. Hence there is great concern to detect such damage and assess whether it is sufficiently extensive so as to result in the structure losing ultimate, and even limit, load capability.

Due to the high relevance of this issue, much research work has focused on the detection of impact damage in composite structures using fibre optic sensors. Whilst the use of advanced processing techniques for networks of multiple FBG sensors will be treated in Section 4.6, and that of Lamb-waves-based methods in Section 4.8, in this Section some recent work focusing specifically on impact damage detection will be reviewed.

One possible technique to monitor the occurrence, energy and position of impact is based on the detection of shock waves with surface-mounted FBG sensors and high speed interrogation systems. L.K. Cheng *et al.* [134] were among the first to use this method in 2002. A plastic round was equipped with two FBG sensors. Impact induced shock waves were generated by dropping a steel ball or a hammer onto the top surface of the plastic rod. Using a sampling frequency of approximately 19 kHz, the shock wave was observed clearly and its propagation speed calculated.

In [135], Kosters *et al.* used the same detection method and developed a system specifically for the SHM of composite primary aircraft structures, capable of interrogating simultaneously 32 FBGs (eight sensors per channel), each at the maximum sampling frequency of 19.3 kHz.

Initially a quasi-isotropic carbon fibre reinforced plastic (CFRP) plate was fabricated and equipped with surface-mounted FBG sensors close to the edges of the plate for an impact hammer test. The impact energy related to the impact event could be inferred from the sum of the weighted sensor output amplitudes. Instead the location of the impact location was estimated from the difference between the arrival times of the impact-induced shock waves at the different sensor locations. In this way, it proved possible to identify impact location with an accuracy of less than 10 cm. Then a second experiment was performed on a composite wing-control surface element, consisting of an upper and lower skin plate, four ribs, two spars and a folded plate normally used at the trailing edge. Four FBG sensors were positioned at the specimen edges and a metal object dropped from a defined height in order to hit the top plate; varying the weight and dropping height of the object made it possible to investigate different values of impact energy and velocity. In some cases, the accuracy of the impact location was larger than 10 cm due most probably to the anisotropy of the material.

Since 1998 Tsutsui *et al.* [136,137,138] have been developing an impact damage detection (IDD) system based on the use of both embedded multi-mode and single-mode small diameter optical fibres. The system detection capability relies on the optical loss due to bending of the structure and the embedded fibre after an impact event. It has been found that the maximum amplitude of optical loss in a multi-mode fibre is related to the impact load. In parallel to the use of multi-mode optical fibres, the authors have used several small-diameter FBG sensors to estimate the impact location from the difference in time of arrival of the shock waves to the sensors locations. In [136] a stiffened composite panel was instrumented to investigate the occurrence of defects below the mid-bay skin and stiffeners. By changing the impact position and load, it was possible to correlate the normalized optical intensity in the surrounding multi-mode fibres to the impact load. When a BVID occurred while increasing the impact load at a certain location, the normalised intensity dropped suddenly and rapidly, appearing as a discontinuity in the plot of the impact load time history. The variation of the measured strain with time experienced a quite similar discontinuity. In correspondence of that discontinuity, it was possible to evaluate the BVID position from the difference in the time of flight of the shock wave to each sensor location.

Recently, Vella *et al.* [133] used the previously mentioned full-spectrum interrogator of fibre Bragg gratings at 100 kHz for the detection of impact loading, improving a previous version of the system that demonstrated full-spectrum FBG interrogation at 535 Hz [139] based on the use of a high-speed tunable optical filter, parallel processing using a field-programmable gate array (FPGA) and Ethernet communications.

The maximum full-spectrum acquisition rate proved suitable to capture the data during low-velocity impact events and transient strain distributions due to openings in delamination phenomena. The significant increase in the reading rate was made possible by driving the tunable filter with a sinusoidal voltage, recording the power as a function time and post-processing the data to obtain a time-varying spectrum rather than processing it in real-time. Details of the system operation can be found in [133]. The authors then used the system to detect impact in a 24-layer composite specimen with an FBG sensor embedded at mid-plane, 16 mm from the impact location. The specimen was impacted repeatedly until perforation. Interrogation of the FBG was performed after each strike at 100 kHz, with a wavelength resolution ranging from 28 pm near the centre of the spectrum to 5 pm at the edges.

**Figure 17:**
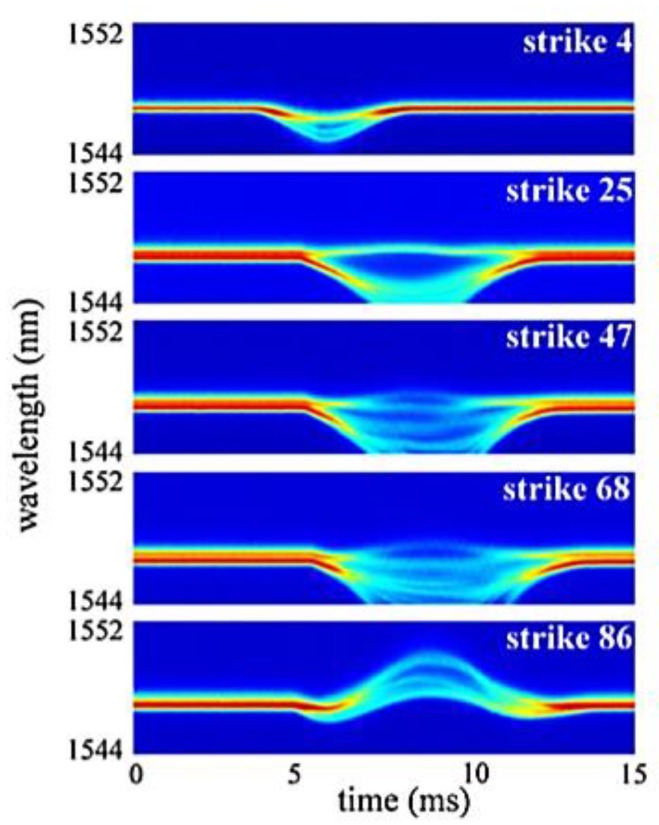
Full-spectral FBG response after different strike numbers [133].

The results are shown in Figure 17. It can be seen that the impact causes compression of the fibre while later the fibre appears to be tensioned. In both cases, the time needed to release the strain state is increased and the spectrum becomes wider as the material approaches failure. Without recording the full spectra it would have been impossible to register the broadening of the FBG reflection length and the splitting into multiple peaks. Instead, if only the most significant peak had been tracked, only a small change, in the order of 1 nm or less, would have been noticed.

Nevertheless, it is necessary to bear in mind that the use of the Bragg spectrum, and the modifications to its shape occurring in case of damage, requires the optic fibre sensor to be in close proximity to the impact location. Therefore, to use such a method, the deployment of a large number of FBG sensors over the area to be monitored is necessary.

### 4.6. Damage Detection Using Advanced Pattern Recognition Techniques

In order to estimate more precisely the location of possible defects such as impact damage, delamination *etc.* using dynamic strain measurements obtained with a network of FBG sensors, research has focused on the use of advanced pattern recognition techniques, based on independent component analysis (ICA) or principal component analysis (PCA) and specific indices.

**Figure 18:**
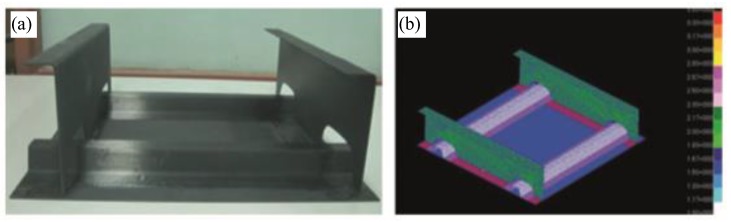
(**a**) Composite specimen representing a typical aeronautical construction; (**b**) FE model of the specimen [140].

In [140,141] the authors used FBG sensors to perform dynamic strain measurements, feeding them into a neural network in order to identify damages in a composite specimen which represented a typical aeronautical construction consisting of skin, frames and stringers (see Figure 18). Also a finite element (FE) model of the specimen was developed in order to simulate its dynamic behaviour. From the analysis of the numerical results, it was verified that the acquisition rate of the available interrogator, *i.e.*, 1 kHz, was sufficient to capture the specimen dynamics using FBG sensors.

Furthermore the optimal locations for the sensors were evaluated so as to be able to measure the highest strain values for the greatest number of mode shapes. Based on these results, three sensors were surface mounted on the frame and one on the skin. An excitation force generated using a frequency sweep in the 0–500 Hz range was then applied to the specimen by using an electromechanical shaker. Different damage scenarios were simulated by attaching six lumped masses in six different areas of the test structure; the number and weight of the masses were changed to represent different damage cases. The results obtained from the four FBG sensors in terms of strain time signals up to 500 Hz were used for modal identification. The frequency spectrum was calculated, and the modal parameters extracted, and were found to be in close agreement with those estimated using the FE model and measured by a reference accelerometer. However, no significant difference between the various damage scenarios was exhibited. Indeed, the variation of the modal parameters such as the modal frequency or amplitude was too limited to detect small defects, making this approach rather useless in many practical situations. To overcome this limitation, the authors developed a method based on advanced pattern recognition techniques. Firstly, a series of parameters was extracted from the time signals in the time, frequency and combined time-frequency domains, using discrete wavelet transforms. Then, feature reduction was performed using the independent component analysis (ICA) technique to generate a new set of parameters, statistically independent from the original ones. Finally the resulting set of features was processed using a support vector machines (SVM) method to make a classification of the data and distinguish between the different damage states. However, for the validation of the method, only one added mass was considered in each damage scenario. In this case, the results were highly encouraging, demonstrating that, after training, the system was able to recognize with high reliability the presence of the single damage and its position on the structure.

In 2013 Sierra *et al.* [142] used a similar approach: Instead of ICA and SVM, their method was based on the principal component analysis (PCA) technique and the use of different damage indices. In addition, in [143] more complex damage scenarios involving more than one defect simultaneously were analysed. To increase the sensitivity and reliability of the method, suitable classification and clustering techniques and the “Optimal Baseline Selection” methodology were proposed. The automatic classification technique based on Self-Organizing Maps (SOM) and Local Density-based Simultaneous Two-Level SOM (DS2L-SOM) algorithms proved to be highly accurate and promising in terms of classifying the data.

### 4.7. Damage Detection in Advanced Grid Structure (AGS)

**Figure 19:**
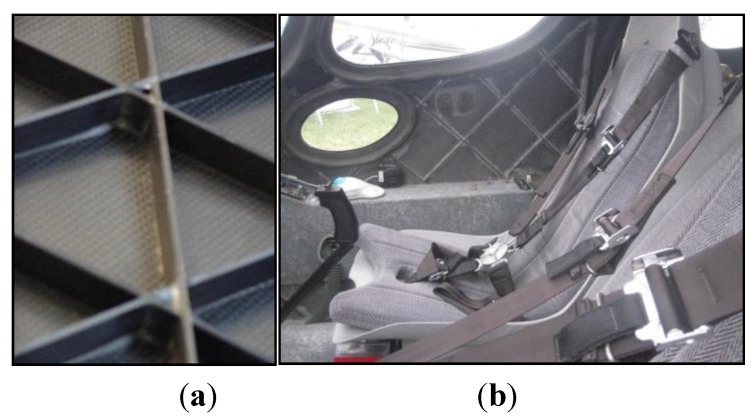
Example of advanced grid structure made of CFRP (**a**) and its application to an aerospace structure (**b**).

One specific, typical aircraft structure, to which FBG sensors may be successfully applied for damage detection, is the advanced grid structure (AGS) made of carbon fibre reinforced plastic (CFRP). AGS (see Figure 19) is often employed in aerospace structures since the ribs effectively carry the axial forces along the direction of the carbon fibre, and the grid itself is lightweight and damage tolerant due to the redundancy of rib elements. Amano *et al.* [144] developed a SHM system for AGS based on FBG sensors embedded in the ribs to monitor longitudinal strain and detect possible failure. One FBG sensor was integrated into in each rib, taking advantage of the fact that the material is unidirectional and therefore the fibre can easily be aligned parallel to the carbon fibres without affecting mechanical properties; moreover, strain is only longitudinal in the ribs.

**Figure 20:**
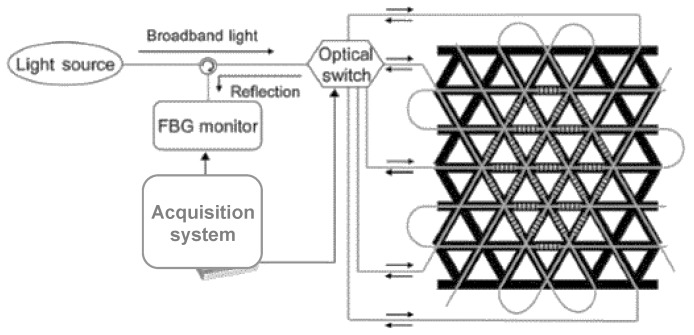
Advanced structural grid (AGS) with SHM system (adapted from [144]).

Preliminary low-energy impact tests were conducted to analyse statistically the failure modes in the grid structure, the results demonstrating that damage phenomena affect only the ribs and not their intersections, and that the primary failure mechanism, in the case of low-energy impact, consists in the micro buckling of carbon fibres. An improved finite element model was also developed in order to determine the best indicator for the presence of damage in the ribs. It was found that the change in static strain at the rib level in the carbon fibre direction is the most sensitive signal to rib cracking. Therefore, a significant and detectable difference between the strains in damaged and undamaged grid structure, Δε, is able to provide precious information regarding the health status of the ribs. In order to determine whether or not these differences are actually significant with respect to the normal expected values, the authors applied a statistical method based on a discordancy test. The SHM system was verified on an advanced structural grid with a network of embedded FBG sensors, as shown in Figure 20. This measurement scheme was proposed originally in [145]. Each instrumented rib had two FBG sensors integrated in the material and an electrical strain gauge mounted on the lower surface. Overall, 19 FBG sensors were deployed in the AGS, each at the midpoint between two intersections.

The first verification test consisted in the application of a three-point bending loading at a velocity of 1 mm/s. Data from the FBG sensors was gathered using an optical spectrum analyser with 10 pm wavelength resolution. Results from the optical and electrical strain gauges agreed reasonably well, demonstrating the reliability of the embedded FBG sensors in measuring the correct strain values in the ribs.

The second verification test was conducted after two of the ribs were notched, approximately at the midpoint between the FBG and the nearest intersection. This time the load was applied in one point of the composite grid and the difference between strains in the damaged and undamaged case, after load application, was used according to the discrepancy test. The strain difference measured by the FBG sensors proved clearly the existence of damage in the notched ribs, even if it was only possible to provide approximate information concerning the damage location.

### 4.8. Damage Detection Using Lamb Waves

Since their discovery by Lamb in 1917 [146] and their first application to nondestructive testing in 1957 [147], Lamb waves have been increasingly used in several nondestructive testing (NDT) applications, included aerospace composite structures [4]. Lamb waves are generally known as “plate waves” and are elastic waves that can be generated in a solid plate with free boundaries. Their propagation can be parallel and perpendicular to the plane of the plate, and their motion can be symmetrical and anti-symmetrical with respect to the neutral axis of the plate. Various techniques exist to generate and receive Lamb waves, but the most common are based on the use of piezoelectric probes with their axis normal to the plate surface.

In recent years, FBG sensors have been applied with success as receivers of Lamb waves instead of piezoelectric probes [148,149]. In this case, the detection is based on the change in grating pitch caused by the propagating elastic waves. The corresponding Bragg wavelength shift allows reconstruction of the received Lamb waves. The combined used of piezoelectric actuators and FBG sensors makes it possible to develop hybrid SHM systems in which the optical sensors monitor the interaction of Lamb waves with the defects. Potentially this type of system is able to detect, localize and evaluate in-service damage in composite structures, with the advantage given by the possibility of embedding the sensors.

In order to monitor Lamb waves, the grating length of the FBG sensor should not exceed the wavelength of the propagating wave which would in fact cause non-uniform strains and therefore broadening of the spectrum width; beyond requiring difficult interrogation, the distortion of the spectrum would give ambiguous information [150]. Different studies have been reported in the literature to identify the best ratio between the grating length of the FBG sensor and the wavelength of the Lamb waves [84,148,151]. The authors of these studies suggest to use a value of 1/6 [151] or 1/7 [84] to obtain the best signal-to-noise ratio; or to use a higher value, between 1 and 4, for more common applications, depending on the required sensitivity [45].

Various works have investigated the use of such hybrid systems for structural health monitoring purposes. In [152] Ogisu *et al.* used and hybrid system with lead zirconate titanate (PZT) actuators and FBG sensors and develop a high-speed interrogation system using an arrayed waveguide grating (AWG) filter to detect disbonds in CFRP box structures. They embedded small-diameter FBG sensors in the bonding areas of the CFRP structure to monitor them during fatigue testing. Disbonds were detected by conventional ultrasonic inspection and from changes in the transmitted Lamb waves. Time signals measured with the AWG-based interrogation system showed that before the occurrence of debonding, two modes could be clearly seen in the plots and that another mode appeared between the first two while the disbond length increased. A wavelet transform algorithm was applied to better highlight the change in the waveforms due to the presence of disbonds [84]. Two parameters were introduced to evaluate qualitatively this waveform change: the damage index DI and the correlation coefficient c. The first was based on the difference between wavelet coefficients of the standard and compared data; the second was equal to the correlation coefficient of the wave envelopes between the two sets of data. More details can be found in [153,154].

Tsuda [155] and, later on, Lam *et al.* [156] proposed a different interrogation architecture using a secondary FBG filter cascaded with the FBG sensor. This architecture used a broadband source (BBS) with two FBG sensors. The first FBG caused a phase shift in the reflected spectrum that intersected with the spectrum of the second FBG sensor. However, this architecture required the center wavelength of the two FBGs to be within close range for successful operation. Using the cascaded FBG interrogation architecture, Tsuda [155] was able to detect a damaged area of 65 mm × 15 mm. More recently, the work of Lam *et al.* [156] reported a detected delamination of 20 mm in a composite beam.

In 2013, Tan *et al.* [157] investigated damage detection and localization in an aluminum plate using an algorithm based on the time of flight (ToF) obtained from four different FBGs. The detectable damage size threshold improved, reaching 6 mm.

More recently, in 2014, Barazanchy *et al.* [12] developed a hybrid system with a piezoelectric actuator and FBGs to detect and localise a through thickness damage (a hole of 2 mm diameter) and a surface damage (a hole with 2 mm diameter and 0.65 mm depth) located on an aircraft representative CFRP plate skin. The use of an elliptical triangulation algorithm [157] allowed the authors to detect damages as small as 2 mm in diameter, with a lower number of sensors and outside the area enclosed by them, unlike previous demonstration of hybrid SHM systems with FBG and piezoelectric transducers. A scenario of this type is more realistic in the case of large aerospace structures and for their health monitoring under operating conditions. To interrogate the FBG sensors, the authors used a tunable laser interrogation system that in a previous study [158] gave the best results in terms of accuracy and repeatability with respect to other architectures. The proposed detection system was based on the faster fundamental symmetric mode (*S*_0_ with a group velocity of 400 m/s) which was not significantly affected by interference with the slower fundamental antisymmetric mode (*A*_0_, 2800 m/s). Based on the estimated dispersion behavior, the selected frequency range for the *S*_0_ mode was between 50 and 400 kHz. The FBG sensors and piezoceramic disc actuator used in the study were surface mounted on the plate.

Considering the selected frequency range and the actuator diameter (6.35 mm), it was decided to generate *S*_0_ waves at a frequency of 325 kHz, which corresponded to a velocity of 4800 m/s and a wavelength of approximately 15 mm. The length of the FBG sensors was selected based on the previously mentioned work by Takeda *et al.* [84] demonstrating that 1/7 is the best ratio between the sensor length and the wavelength of the Lamb waves to be detected. However, due to availability of sensors, 5 mm long FBGs were actually used. The interrogation of the FBG sensors was based on the use of a fine linewidth tunable laser, an oscilloscope with an acquisition rate of 125 MS/s and a photodetector connected to the oscilloscope. Prior to each actuation, the laser was tuned to the wavelength of one FBG sensor. This operation was repeated for all sensors within one scan. The data were then post-processed using an algorithm developed for damage detection and localization, which was based on the time of arrival (ToF) of the highest signal peaks after actuation. Travelled distances were then calculated starting from the ToF and the *S_o_* wave velocity at 325 kHz, the distance travelled by the actuated propagating wave being the sum of the distance from the actuator to the damage and that between the damage and the detecting FBG sensor. This means that all possible damage positions lay on ellipses with focal points at the actuator and sensor locations [157]. A “ring” representing all possible damage locations was obtained for each actuator sensor pair by reconstructing the ellipse equation for both the velocities.

**Figure 21:**
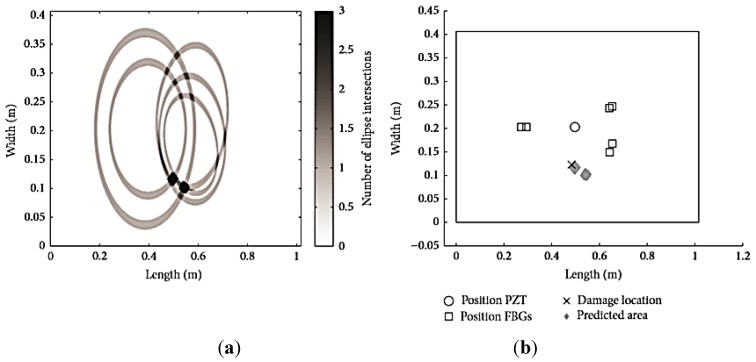
(**a**) Intersection of the calculated ellipses; (**b**) comparison between the actual and predicted damage location [12].

In this operation, the only unknown variable was the length of the minor axis of the ellipse. By repeating this process for all the actuator-sensor pairs, the probable damage location on the plate could be associated with the area where most of the rings intersected (see Figure 21a).

The algorithm was applied to the detection of the mentioned defects for verification, and the results for the case of the through thickness hole of 2 mm are shown in Figure 21b. The estimation error was about 5 mm, and in the case of the surface damage hole, the position was estimated with an error of 25 mm. Hence, with a relatively simple algorithm based on a standard signal processing and localisation technique, it was possible to obtain very promising results.

### 4.9. FBG Sensors in Smart Composites

In recent years, the use of shape memory alloy (SMA) wires integrated into composite structures to create “smart composites” has attracted much research work owing to their capability of providing control on the shape and mechanical properties of composite structures [159]. SMAs are materials with the ability to return to their original geometry at characteristic temperatures following large inelastic deformations. An increase in temperature allows their shape recovery also under high loads, which results in high actuation energy density [160]. The actuation mechanism of SMAs can thus be controlled by the heating process. In this case, the recovery stress is much higher than the stress required to pre-strain the material at lower temperatures.

The operational effectiveness of SMA actuators in a smart composite is dependent on the interfacial bonding between the embedded SMA wires and the surrounding matrix. Although wire twisting and surface heat treatment methods can be used to enhance the bonding quality of SMA wires, poor bonding performance affects strain transfer and increases the possibility of debonding. Therefore, an effective sensor technology for monitoring the correct operation of SMA actuators in smart composites is highly desirable. In this respect, embedded FBG sensors have been shown to be a promising option, as they are able to provide static and dynamic strain information on the SMA actuators operation [159].

FBG sensors can also be employed beneficially when alternative concepts for morphing composite structures using SMAs are considered, without the actual embedding of the actuators in the material. In this case, e.g., a wing only partially made of composite, FBG sensors allows monitoring of the composite element subjected to shape change.

The application of SMAs to aircraft structures [161] includes mainly the possibility of realizing the so-called “adaptive wing” in which special actuators are used to modify the shape during flight, and control its aerodynamic and flight dynamic characteristics. In recent years, with the massive introduction of composite materials in the aerospace industry, research has focused on the potential development of morphing lifting composite structures and the monitoring of their operation and structural health. Shape memory alloys are considered to be a viable solution to introduce the desired geometry change to fit the current flight conditions without suffering critical deformations, especially in lightweight aircrafts, such as unmanned aerial vehicles. To date, SMAs have been used mainly for twist and camber morphing [161]; however, more research is still needed to address some concerns regarding their limited fatigue life and slow dynamic response [161]. In parallel, effective ways to control the operation of SMA actuators have been researched, focusing on the use fibre optic strain sensor technology.

**Figure 22:**
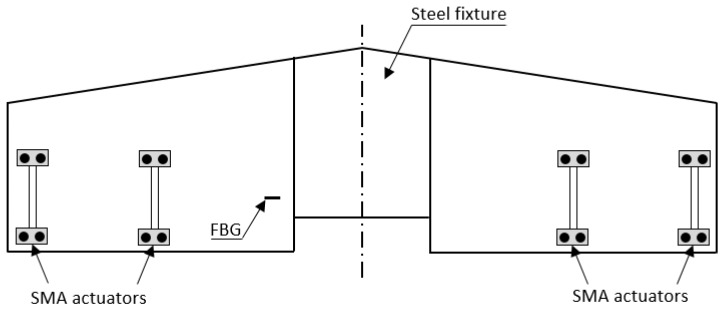
Scheme of the smart composite wing experiment (adapted from [162]).

In [162] Yang *et al.* investigated a morphing wing with variable camber using FBG sensors. The wing consisted of two graphite/epoxy composite skins and a steel supporting frame. The SMA wire actuators were attached to the bottom skin, and the FBG sensors were surface-mounted close to the wing root (see Figure 22) in order to measure the dynamic strain during actuation of the wires. Lift and drag forces were measured at different angles of attack to investigate the aeroelastic phenomena and their mitigation via the use of SMA actuators.

The smart composite wing was then statically and dynamically tested in a subsonic wind tunnel, using a supporting frame instrumented with a 6-axis load cell. Signals from the FBG sensors were acquired by using an FBG interrogation system based on a wavelength swept fibre laser reaching a speed of 200 Hz. In particular FBG sensors were used to investigate flutter phenomena and the effect of SMA actuation on them. Flutter modes were firstly estimated at different airflow velocity and angle of attack; then the amplitude of the limit cycle oscillation was measured again after application of the electric current to the SMA wires. As a result of SMA actuation, the transverse stiffness of the wing increased, thus significantly reducing the amplitude of oscillation at certain values of the applied electric current. FBG sensors successfully enabled the monitoring of SMA actuators effectiveness on the mitigation of flutter phenomena under different operating conditions. This concept might be applied not only in laboratory experiments but also for real-time monitoring of the morphing wing during flight.

In 2011, Mieloszyk *et al.* [163] developed a SHM system based on the use of FBG sensors to monitor shape and defects in an adaptive wing with composite skin; a wing prototype with a span of 1 m was manufactured consisting of five sections, with four of them allowed to rotate around a common rod. The rotation was realised using SMA actuators integrated at the points connecting the sections on the supporting rod. The outer composite skin was instrumented with a surface-mounted array of four active FBG sensors and one temperature-compensating sensor. Two main types of scenario were considered for monitoring of the wing morphing properties: One in which the twisting moment transmitted by a SMA actuator was reduced, as if the actuator was flawed; and a second in which a skin damage was introduced. The presence of damage in this case is seen as a factor limiting the morphing properties of the wing. In order to assess whether the SMA actuator was transmitting the expected amount of torque to the wing section, the authors adopted an approach based on artificial neural network (ANN). In the second scenario, a series of small masses was added in various positions on the top skin of the wing to simulate local icing. The related results and procedure are detailed in a previous study reported by the authors in [164]. In this case as well, the strains obtained from FBG sensors were input into a neural network based system, which allowed damage localization in a particular segment of a wing section (leading edge, middle line or trailing edge) with a 100% high level of confidence.

A quite different application of FBG sensors used in conjunction with SMA wires is reported in [165,166]. In these works, the authors developed a “smart crack arrester” for composite sandwich structures based on the crack arrester proposed by Hirose *et al.* [167], which consisted of a semi-cylinder element of stiff material inserted beneath the facesheet. The aim of the arrester was to decrease the energy release rate of a crack when its tip approaches the arrester by suppression of the local deformation. In [165] the authors added both SMA wires and FBG sensors to the stiff element in order to provide a useful memorizing and detection function. Two metal wires and two sensors were embedded at both edges of the arrester. In this way, the crack energy release is transmitted to the SMA wire and the related residual strain is detected by the FBG sensor through the birefringence effect experienced by the fibre in presence of transverse strain. The detection capability allows appropriate measures to be taken for maintenance of the damaged part.

## 5. Applications of Distributed Fibre Optic Sensors

### 5.1. Strain-Based Shape Reconstruction

With respect to multi-point techniques that use FBG sensors for strain-based shape reconstruction [109,110,111,112,113,114,115,116], the main advantage offered by distributed sensing is the large availability of strain data due to millimeter resolution over long fiber spans. This approach can therefore improve substantially the application of algorithms used for shape reconstruction starting from strain measurements.

**Figure 23:**
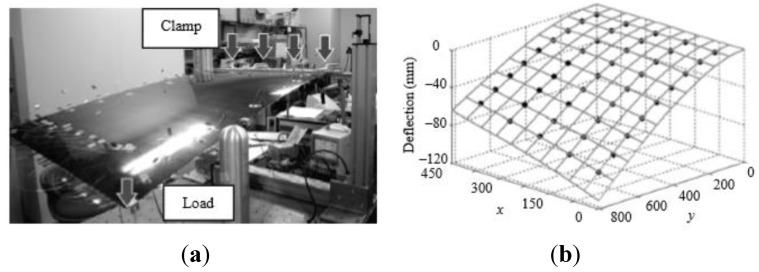
Photo of the cantilever plate experiment (**a**); reconstructed deflection shape of the plate (**b**) [166].

In [168,169] a modal-based algorithm was used for shape reconstruction using strain measurements obtained by the Pre-pump pulse Brillouin Optical Time-Domain Analysis (PPP-BOTDA) technique. The algorithm was applied to the composite specimen shown in Figure 23a. The specimen was clamped and subjected to a load applied at the tip, and the estimated deflections were compared to those measured using a laser displacement sensor, exhibiting a good agreement (see Figure 23b. The use of this approach also made it possible to take into account existing non-uniformities in the measured strain profiles, thus improving the accuracy of shape reconstruction.

### 5.2. Strain Monitoring in Structure Elements

**Figure 24:**
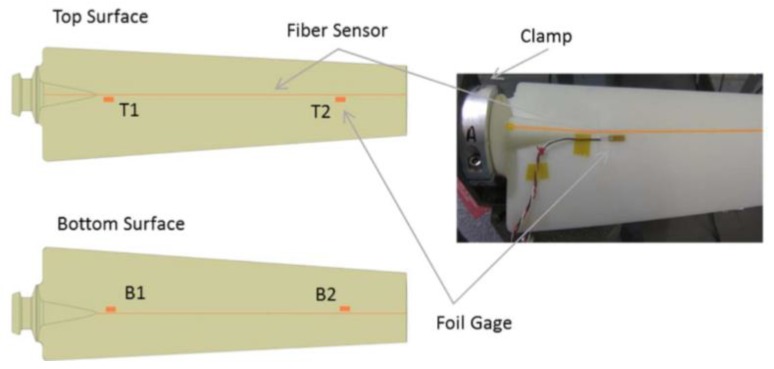
Position of the optical fiber and foil gage in the clamped fan experiment [65].

Recently, the acquisition rate of distributed systems has been improved, opening up new possibilities for application to aircraft structures. In 2013 Rahim *et al.* [65] performed dynamic strain measurements on a one meter composite axial fan blade using the Optical Frequency Domain Reflectometry (OFDR) technique based on Rayleigh scattering, acquiring strain data with a two-meter long standard telecom optical fiber at the rate of 250 Hz. The fiber was polyimide-coated, with a diameter of 155 µm, bonded to the top surface of the blade which was clamped at its root, as shown in Figure 24. The rest of the fiber was bonded to the bottom surface, and four electrical strain gauges were located close to the fibers (see Figure 24. The clamped blade was mounted on a shaker and oscillated at an acceleration level of 1G using a frequency sweep from 5 to 120 Hz. In a second dynamic test, a point mass of 80 g was added to the blade tip.

A commercial system capable of interrogating up to 10 m of fibre was used to measure the static and dynamic strains with a spatial resolution of 5 mm. The blade was also simulated using a FE model. In order to compare simulated and experimental data, strain measurements were converted to mode shapes. During dynamic testing, measurements from the fibre sensor and the foil gauges exhibited good agreement, with only a 3.25% absolute difference being observed for a frequency sweep from 5 Hz to 120 Hz. Good agreement was also reported between the mode shapes. The high spatial resolution enabled the comparison of experimental and computed displacements over the entire fibre length rather than at discrete points. Furthermore, analysis at an acquisition rate up to 250 Hz would be feasible. These interesting results indicated the potential application to large complex composite structures for model validation and structural health monitoring, such as operational load monitoring, fatigue monitoring and strain-based shape reconstruction.

### 5.3. Detection of Damage in Joining and Bonded Elements

**Figure 25:**
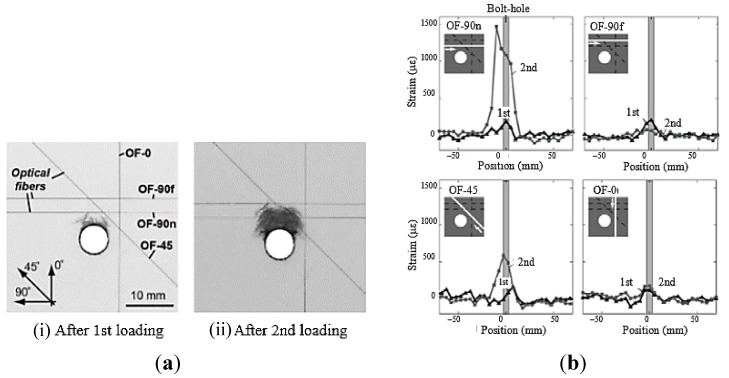
Optical fibres deployment and damage around the CFRP bolted joint (**a**); residual strain distribution along the optical fibres after loading (**b)** [166].

Minakuchi *et al.* [170] used a Brillouin Optical Correlation Domain Analysis (BOCDA) system to detect bearing failure in CFRP bolted joints, key elements of aircraft structures. As reported in Section 2, BOCDA is a distributed sensing system with millimetre-order spatial resolution developed by Hotate *et al.* [77,78,79]. Optical fibres were embedded along bolt holes, as shown in Figure 25a, and the variation in strain distribution enabled the monitoring of bearing failure.

The deployment of the fibres was based on a preliminary investigation of damage modes occurring after failure and on a feasibility study by a finite element analysis model simulating damage propagation and residual strain change [171]. Figure 25b reports also typical plots representing the residual strain variation measured along the fibres: it can be seen that the system was able to capture permanent strain variation due to fibre micro-buckling (kinking) damage or residual deformation of the surrounding plies. From the strain distribution pattern with millimetre resolution, it was also possible to evaluate the damage size and direction, hence indicating the potentially applicability of such a system to large-scale composite structures carrying a high number of bolted joints.

The detection of debonding in structural elements, such as stiffeners for example, is another potentially attractive field of application for distributed sensing systems: Indeed, areas between bonded elements can be instrumented with optical fibres over their whole length. In [172] Minakuchi *et al.* embedded a single optical fibre between the stiffeners and the skin of a CFRP panel, and were able to estimate effectively the residual strains after manufacturing and the damage which occurred during impact tests using the Pre-pump pulse Brillouin Optical Time-Domain Analysis (PPP-BOTDA) technique. More recently, Guemes *et al.* [173] conducted a similar experiment by using instead an OFDR system based on Rayleigh scattering, investigating in particular the progressive debonding introduced by subjecting the stiffener to bending at one edge. In a following test, a BVID was introduced very close to the stiffener. In both cases, the strain maps measured with the OFDR system clearly indicated the areas where strains had changed due to the presence of damage.

### 5.4. Detection of Impact Damage

In [173,174] Guemes *et al.* reported a series of experiments on CFRP panels to detect delamination caused by impact. In this case, the advantage offered by distributed sensors is the possibility of creating a very high-density sensor network with a single optical fibre. By deploying the optical fibre over the entire structure, and taking care to avoid sharp curvatures which may cause significant losses of light, a sensing grid can be created to monitor a wide structural area with spatial resolution down to a few millimetres. Even without a-priori knowledge of the damage location, the chances of detecting it are increased substantially with respect to the use of quasi-distributed techniques which also require more complicated schemes of detection based on multiplexing techniques. Strain measurements obtained from a full area can then be used to create a map of strain distribution. Each time a damage causes a change in the strain field, it is possible to visualise it through images representing the strain level over the structure and its local gradients. In [174] the authors embedded optical fibres in CFRP laminates and measured the change in the strain measured after impacts with increasing levels of energy.

**Figure 26:**
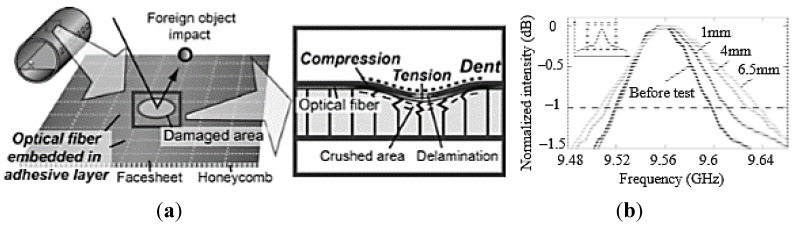
Impact test layout (**a**); BGS measured in the point closest to the impact location (**b**) [166].

In [105] Minakuchi *et al.* applied a Pre-pump pulse Brillouin Optical Time-Domain Analysis (PPP-BOTDA) system to the investigation of impact damage on composite sandwich structures, exploiting the enlargement of the Brillouin gain spectrum (BGS) due to non-uniform strain along the fibre. In general, the central frequency of a measured Brillouin gain spectrum (BGS) is related to the average value of axial strain over the sensing system spatial resolution. Thus, the strain variation in a damaged area smaller than the spatial resolution hardly changes the BGS peak frequency. However, the BGS shape contains information regarding the strain distribution within the spatial resolution [175] thus making the system potentially sensitive to small damages. For the verification tests, the authors used the experimental layout shown in Figure 26a.

A grid of optical fibres was embedded in the adhesive layer between the composite facesheet and the lightweight core in order to measure strain variation at the interface between the two materials. Indentation of the panel surface induces non-uniform tensile and compressive strain in the optical fibre, hence resulting in a variation in the BGS profile: Results are shown in Figure 26b, where the broadening of the spectrum is clearly visible. While the BGS width measured at −1 dB increased in relation to damage growth, the peak frequency remained constant. Hence the BGS central frequency was unable to reveal the defect, whilst examination of the spectrum width could enable the detection of the presence of a barely visible damaged area. The authors used this technique to monitor also the accumulation of water in honeycomb sandwich structures [176].

### 5.5. Novel Concept for Distributed Sensing

In order to be fully mature for the structural health monitoring of aerospace structures, critical issues such as robustness and maintainability need to be addressed in particular as regards the systems based on fibre optic sensors described so far in this paper. For example, replacing a damaged fibre is relatively complicated, especially if embedded. In order to overcome these problems and to increase the spatial resolution, in [8] the authors proposed a “hierarchical fibre-optic-based sensing system” similar to the nervous system in vertebrates. In such a system, several types of specialised devices are hierarchically combined to form a sensing network. Specifically, numerous three-dimensionally structured sensor devices are distributed throughout the whole structural area and connected by an optical fibre network, which is not embedded into the structure. Since the optical fibre is attached to the back surface of the structure, the fibre rarely breaks when the structure is damaged. In the case of breakage, the damaged part can be replaced more easily using a fusion splicer. Furthermore, in the proposed system several monitoring sensors connected to different optical fibres are located in the same area thus providing redundancy and therefore robustness. In order to validate the system concept, an impact damage detection system was developed based on a hierarchical sensing architecture. Surface-crack sensing devices based on comparative vacuum monitoring (CVM) sensing devices were used and monitored by an optical fibre network. This system was applied to a CFRP skin-stringer fuselage demonstrator, and barely visible impact damage (BVID) was successfully detected from a strain increase in the optical fibre in the damaged area.

## 6. Conclusions

In this paper, recent advances and applications of FBG sensors, Brillouin and Rayleigh distributed sensors to the structural health monitoring of composite aircraft structures have been reviewed. The performance characteristics and intrinsic limitations of currently available fibre optic sensors and systems have been discussed with reference to the basic principles and the specific technologies of sensors and their sensor deployment in composite structures and possible application cases.

A number of systems that have been proposed to date have been demonstrated to be already technologically mature for application in real situations, such as strain-based shape and operational load monitoring, included in smart composites (based on shape memory alloys, SMA, for example), or the detection of disbonds or damage in composite repair patches, lap joints, bonded or joining elements. In these cases, distributed sensing may already offer several advantages over quasi-distributed sensorsin situations in which large rather than local areas are to be monitored, with high spatial resolution at relatively low frequency. In other cases, particularly the detection and evaluation of damage caused by impact, the location of which is difficult to determine in advance, there is much promising ongoing research focusing on the joint use of dynamic strains measured with FBG sensors and advanced pattern recognition algorithms, such as those based on principal component analysis (PCA) or independent component analysis (ICA) techniques, or piezoelectric sensors to generate Lamb waves. These SHM systems offer the possibility of monitoring the presence, size and progression of small impact damage (currently in the order of millimetres) in real time, in flight, and also on demand in the case of piezoelectric actuators. In this respect, distributed sensing systems are currently limited by the relatively low acquisition frequency allowed by the technology; their use for impact damage detection would need to be based on a different strategy, such as the deployment of a network of optical fibres which would enable the measurement of static strain changes with high spatial resolution, thus increasing the chances of detection in close proximity to the damage area.

To achieve the ultimate goal of establishing a standardisation framework for sensor quality, application and integration in the aircraft environment, as envisaged by Airbus in the forthcoming years, some additional research and development are required to ensure highly reliable detection capability, robustness in order to avoid signal loss in the case of fibre breakage, and maintainability to permit the replacement of damaged fibre sensors.
